# Surface protein profiling reveals depot-specific features of subcutaneous and visceral adipose progenitor cells

**DOI:** 10.1016/j.bbrep.2026.102750

**Published:** 2026-08-17

**Authors:** Kazuki Hachiya, Nobuo Yamasaki, Hiroto Fukai, Yuki Uchida, Yuka Nozaki, Yuhei Mizunoe, Yoshikazu Higami

**Affiliations:** a Laboratory of Molecular Pathology and Metabolic Disease, Faculty of Pharmaceutical Sciences, Tokyo University of Science, Tokyo, Japan; b Research Institute for Biomedical Sciences (RIBS), Tokyo University of Science, Noda, Japan

## Abstract

White adipose tissue is organized into anatomically and functionally distinct depots, yet the mechanisms by which intrinsic differences between subcutaneous and visceral adipose progenitor cells (APCs) contribute to depot-specific inflammatory and remodeling properties remain unresolved. Here, by directly comparing APCs isolated from subcutaneous and visceral fat, we identify distinct surface protein programs underlying their divergent functional properties. Subcutaneous APCs exhibited elevated expression of galectin-9, CD44, and CD63, defining functional programs governing immune modulation, adipogenesis, and exosome-mediated stress adaptation. Functional assays revealed that galectin-9 promotes an anti-inflammatory phenotype, CD44 quantitatively modulates adipogenic differentiation, and CD63 enhances exosome release and confers resistance to iron-induced cellular stress. Notably, several depot-specific features were not evident at the transcriptomic level, underscoring the importance of surface protein-level regulation. Together, our findings demonstrate that APC surface protein signatures distinguish subcutaneous from visceral fat, establishing a mechanistic link between depot identity, tissue remodeling, and metabolic disease susceptibility.

## Highlights

• Surface profiling reveals depot-specific signatures in subcutaneous vs visceral APCs.
• Galectin-9 promotes an anti-inflammatory phenotype in preadipocytes.
• CD44 levels quantitatively tune adipogenic differentiation capacity.
• CD63 boosts exosome release and protects cells from iron-induced stress.

## 1 Introduction

White adipose tissue (WAT) is an energy-storing organ and an active endocrine tissue that releases diverse adipokines to regulate whole-body energy balance and systemic homeostasis [1]. Through dynamic storage and mobilization of triglycerides, WAT maintains metabolic equilibrium in response to nutritional status [2]. Among the various WAT depots, subcutaneous (sWAT) and visceral (vWAT) are anatomically and functionally distinct, each exhibiting unique metabolic characteristics [[3], [4], [5]]. vWAT is closely associated with insulin resistance, inflammation, and metabolic disease, whereas sWAT plays a protective role by buffering excess lipids and supporting systemic metabolic balance [6]. WAT exhibits remarkable plasticity, expanding or shrinking in response to energy fluctuations through hypertrophy of mature adipocytes and de novo adipogenesis from resident progenitor cells [6].

This dynamic adaptability of WAT depends not only on mature adipocytes but also on diverse non-adipocyte cell populations that orchestrate tissue remodeling. Among these, adipose progenitor cells (APCs), immune cells, and vascular endothelial cells modulate these processes in a coordinated manner. In particular, APCs serve as the cellular source for new adipocytes and are central to WAT renewal [7,8]. Single-cell transcriptomic studies have shown that APCs are not homogeneous; rather, they comprise subpopulations that differ in localization, differentiation potential, and gene expression programs [3,5]. These analyses have further demonstrated that APCs comprise multiple differentiation states along the adipogenic trajectory and that depot-specific transcriptional programs accompany these states. In particular, vWAT-derived APCs (v-APCs) preferentially express pro-fibrotic and inflammatory gene signatures, whereas sWAT-derived APCs (s-APCs) are enriched for genes associated with immune modulation and tissue homeostasis. Importantly, transplantation experiments suggest that these APC properties are primarily shaped by their tissue of origin rather than by the recipient depot, supporting an intrinsic depot identity. Collectively, these findings indicate that APC heterogeneity is strongly depot-specific [5]. This cellular heterogeneity contributes to depot-specific differences in regenerative capacity and inflammatory and fibrogenic responses. Understanding APC diversity is therefore essential for elucidating the mechanisms that govern WAT plasticity and depot-specific function.

In parallel with cellular heterogeneity, adipose depots also exhibit distinct modes of intercellular communication. Extracellular vesicles (EVs) released from vWAT and sWAT carry different molecular cargos, with vWAT-derived EVs enriched in inflammation-related proteins compared with the less inflammatory profiles of sWAT-derived EVs [9,10]. Together, these observations suggest that depot-specific EV signaling reflects intrinsic differences in resident APC populations. Mechanistically, depot-specific differences in adipogenic capacity and inflammatory status have been linked to differential regulation of pathways controlling progenitor fate and tissue remodeling. These include pro-adipogenic factors such as peroxisome proliferator-activated receptor γ (PPARγ) and CCAAT/enhancer-binding proteins (C/EBPs), and anti-adipogenic and pro-fibrotic pathways involving Wnt signaling, hypoxia-associated responses, and TGF-β signaling. Such regulatory networks likely contribute to intrinsic functional differences between sWAT and vWAT depots [6].

Although recent studies have increasingly used the term adipose stromal and progenitor cells (ASPCs) [11] to describe the heterogeneous stromal populations residing in adipose tissue, including adipogenic progenitors, the present study focused primarily on the Hematopoietic lineage negative (Lin^–^)/Sca-1^+^ adipogenic progenitor population. Therefore, for clarity and consistency, we use the term APCs throughout this manuscript.

Despite a growing understanding of depot-specific APC heterogeneity from single-cell transcriptomics, it remains unclear how these transcriptomic signatures correspond to stable, functional differences between APCs. Gene expression profiles alone do not always reflect functional phenotypes, highlighting the requirement for complementary approaches that capture stable, actionable traits. Surface molecules are amenable to isolation and manipulation and represent relatively stable phenotypic features that can link cellular identity to functional behavior through interactions with the tissue microenvironment. Accordingly, we performed a comparative surface-protein screen of APCs isolated from sWAT and vWAT to identify depot-specific molecular signatures. This approach highlighted candidate molecules associated with key functional axes, including inflammatory regulation (galectin-9), adipogenic differentiation (CD44), and exosome-mediated communication (CD63), which we subsequently investigated in detail.

## 2 Methods

**Table:** Key Resource Table

Antibodies		
Reagent or Resource	Source	Identifier
Anti-PDGFRα (FACS)	Invitrogen	Cat# 11-1401-82
Anti-PDGFRα (FACS)	BioLegend	Cat# 135905
Anti-Sca-1 (FACS)	BioLegend	Cat# 122514
Anti-Sca-1 (FACS)	eBioscience	Cat# 11-5981-85
Anti-CD45 (FACS)	Bioss	Cat# bs-4818R-A647
Anti-CD31 (FACS)	BioLegend	Cat# 102416
Anti-Ter119 (FACS)	eBioscience	Cat# 17-5921-82
Anti-galectin-9 (FACS)	BioLegend	Cat# 137903
Anti-CD44 (FACS)	BioLegend	Cat# 103023
Anti-CD63 (FACS)	BioLegend	Cat# 143903
Lineage antibody cocktail	BioLegend	Cat# 133310
Anti-galectin-9 (WB)	Proteintech	Cat# 17938-1-AP
Anti-Alix (WB)	Proteintech	Cat# 12422-1-AP
Anti-CD63 (WB)	Proteintech	Cat# 25682-1-AP
Anti-CD81 (WB)	Cell Signaling Technology	Cat# 10037S
**Experimental Models: Cell Lines**		
**Cell line**	**Source**	**Identifier**
3T3-L1 preadipocytes	RIKEN BRC	RRID: CVCL_0123
Plat-E cells	Gift from T. Kitamura Institute of Medical Science, The University of Tokyo	RRID: CVCL_B488
**Experimental Models: Organisms/Strains**		
**Organism/strain**	**Source**	**Identifier**
Mouse: C57BL/6J	CLEA Japan	RRID: IMSR_JAX:000664
**Chemicals, Peptides, and Recombinant Proteins**		
**Reagent**	**Source**	**Identifier**
Collagenase type II	Thermo Fisher	Cat# 17101015
Hoechst 33342	FUJIFILM Wako	Cat# 346-07951
Ferric ammonium citrate	FUJIFILM Wako	Cat# 158-040
Puromycin	FUJIFILM Wako	Cat# 160-23151
3-isobutyl-1-methylxanthine (IBMX)	Sigma-Aldrich	Cat# I5879
Dexamethasone	Sigma-Aldrich	Cat# D4902
Insulin	FUJIFILM Wako	Cat# 099-06473
Tri-iodothyronine (T3)	Sigma-Aldrich	Cat# T6397
**Software and Algorithms**		
**Software**	**Source**	**Identifier**
GraphPad Prism	GraphPad Software	RRID: SCR_002798
ImageJ Fiji	NIH	https://imagej.net/Fiji
R	R Foundation	v4.5.0
Seurat	Satija Lab	v5.4.0
NanoSight NTA software	Malvern Panalytical	v3.1

### 2.1 Animals

The experimental procedures and reporting of this study were conducted in accordance with the ARRIVE guidelines. All animal experiments and protocols adhered to the Fundamental Guidelines for Proper Conduct of Animal Experiment and Related Activities as stipulated by the Ministry of Education, Culture, Sports, Science and Technology of Japan. The experiments were approved by the Ethics Review Committee for Animal Experimentation at Tokyo University of Science (approval numbers: Y20043, Y21043, and Y22037). C57BL/6 mice were housed in a specific pathogen-free environment and had free access to food, a CRF-1 diet (Oriental Yeast, Tokyo, Japan), and water.

For flow cytometry analysis, male mice were euthanized at 14–16 weeks of age. The subcutaneous sWAT and vWAT were collected.

For RT-PCR, male mice were euthanized at 22 weeks of age. sWAT and vWAT were removed, diced, frozen in liquid nitrogen, and stored at −80 °C until use.

### 2.2 Isolation of adipose-derived stromal cells and surface protein screening

sWAT and vWAT were isolated from adult C57BL/6 mice (14–16 weeks; *n* = 12). The tissues were minced and digested with collagenase type II (1 mg/mL in DMEM) at 37 °C for 1 h with gentle agitation. The resulting cell suspensions were filtered through a 70 μm cell strainer and centrifuged at 300 × g and 4 °C for 10 min to separate the SVF. Red blood cells were lysed using ACK lysis buffer, and the remaining cells were washed with FACS buffer (PBS supplemented with 1% penicillin-streptomycin, 2% FBS, and 2 mM EDTA).

Cells were stained with 7-AAD and antibodies against lineage markers (TER119, CD45, CD31) and Sca-1. Lin– Sca-1+ cells were sorted using a FACS Aria III cell sorter (BD Biosciences). Cell quality was assessed by flow cytometric gating based on viability (7-AAD exclusion) and enrichment of the Lin^–^ Sca-1^+^ population. Freshly isolated cells were used immediately for downstream analyses without cryopreservation. The sorted Lin^–^ Sca-1^+^ cells from sWAT were stained with Hoechst 33342 (5 μg/mL, 30 min at 37 °C) to enable discrimination between sWAT- and vWAT-derived cells after mixing.

Mixed Lin^–^ Sca-1^+^ cells from sWAT and vWAT were plated onto antibody-coated 96-well plates (LEGENDScreen™ Mouse PE Kit; Cat. No. 700009, Biolegend), each well pre-coated with a different PE-conjugated antibody targeting a surface molecule. After incubation, cells were analyzed on a CytoFLEX flow cytometer (Beckman Coulter). Hoechst^+^ and Hoechst^–^ cells were distinguished to assess the differential expression of each surface protein between sWAT and vWAT progenitor populations.

### 2.3 Cell culture and differentiation

3T3-L1 preadipocytes (RIKEN Bioresource Center, Tsukuba, Japan; Cat# RCB2767; RRID: CVCL_0123) were cultured in maintenance medium comprising DMEM supplemented with 10% FBS and 1% penicillin–streptomycin. Cells were obtained directly from the supplier and used within a limited number of passages after thawing. Cell line authentication was performed by the supplier. Cells were tested and confirmed to be free of mycoplasma contamination using the e-Myco™ Mycoplasma PCR Detection Kit (ver. 2.0) (LiliF Diagnostics, Seoul, Republic of Korea). 3T3-L1 preadipocytes were cultured to full confluence and maintained for 2 additional days (designated day 0 of differentiation). Cells were then cultured in adipocyte differentiation medium containing 500 μM IBMX and 1 μM dexamethasone for 2 days, followed by adipocyte maturation medium containing 10 μg/mL insulin and 50 nM T3. The maturation medium was replaced every 48 h, and cells were differentiated for a total of 8 days.

### 2.4 Oil Red O staining of 3T3-L1 cells

Formalin-fixed cells were stained with freshly prepared Oil Red O (Sigma‐Aldrich) staining solution (1.8 mg/mL in 60% isopropanol) for 20 min at room temperature. Cells were washed with 60% isopropanol and H_2_O. The stained cells were visualized using an optical microscope (BZ-X800; Keyence, Osaka, Japan). The obtained images were analyzed using ImageJ/Fiji.

### 2.5 Construction of plasmids for *Lgals9*, *Cd63* and *Cd44* overexpression

Coding regions of *Lgals9*, *Cd63*, and *Cd44* were amplified from 3T3-L1 cDNA using KOD ONE (Toyobo) and suitable primers (Table 2). The amplicons were digested with *Eco*RI and *Xho*I and inserted into the pMXs-AMNN-Puro plasmid [12]. The cDNA sequences were confirmed to match the mouse reference sequences (NM_001159301.1, NM_001282966.2, XM_006498649.2), and the resulting OE plasmids were designated pMXs-AMNN-*Lgals9*-Puro, pMXs-AMNN-*Cd63*-Puro, and pMXs-AMNN-*Cd44*-Puro.

### 2.6 Generation of overexpression 3T3-L1 cells

Galectin-9-OE, Cd63-OE, and Cd44-OE 3T3-L1 cells were generated using retroviral vectors. pMXs-AMNN-puro (mock control), pMXs-AMNN- *Lgals9*-Puro, pMXs-AMNN-*Cd63*-Puro, and pMXs-AMNN-*Cd44*-Puro plasmids were transfected into Plat-E cells (kindly provided by T. Kitamura, Institute of Medical Science, The University of Tokyo, Tokyo, Japan) using PEI MAX (Cosmo Bio Inc., Tokyo, Japan). Twenty-four hours after transfection, cells were washed twice with PBS and restored with fresh culture medium. After 2 days, the supernatant from each virus-containing culture was harvested and filtered through a 0.22-μm filter (Millipore, Billerica, MA, USA). Overexpressing cells were established by incubating sub-confluent 3T3-L1 cells in virus-containing medium for 24 h under standardized infection conditions. Because retroviral particles generated from Plat-E cells were used, an exact multiplicity of infection (MOI) was not determined. Viral transduction was performed using the same culture format and infection duration across experiments, followed by selection with 5 μg/mL puromycin for 5–7 days.

### 2.7 Flow cytometry

3T3-L1 cells were detached using Accutase™ and centrifuged at 300 × g and 4 °C for 10 min. The cell pellets were resuspended in FACS buffer (PBS containing 2% FBS and 1 mM EDTA). PE-conjugated antibodies against galectin-9, CD63, or CD44 were added to the cell suspension and incubated in the dark at 4 °C for 30 min. After incubation, cells were washed, centrifuged, and resuspended in FACS buffer, then passed through a 70 μm cell strainer. Stained cells were analyzed using a BD FACSLyric™ flow cytometer.

### 2.8 RT-PCR

RNA was extracted from tissues and cells using ISOGEN II (Nippon Gene, Tokyo, Japan). Purified RNA was reverse transcribed using ReverTra Ace® qPCR RT Master Mix (Toyobo, Osaka, Japan), and cDNAs were amplified using a CFX Connect™ Real-time System in Thunderbird SYBR qPCR mix (Toyobo) with the primers for each gene, in accordance with the manufacturers’ protocols. Target gene expression data were normalized to *Rps18* expression. The primer sequences used for this study are listed in Table 2.

### 2.9 Western blotting

Cell pellets were lysed in lysis buffer (50 mM Tris-HCl, 2% sodium dodecyl sulfate [SDS], 3 M urea, 6% glycerol, pH 6.8), boiled, and sonicated for 5 min. Protein concentrations of the soluble fraction were determined using the bicinchoninic acid protein assay (Thermo Fisher Scientific, Waltham, MA, USA) according to the manufacturer's protocol and standardized by adding lysis buffer. Protein samples were then mixed with 2-mercaptoethanol and bromophenol blue to obtain final concentrations of 5% and 0.025%, respectively, and heated at 100 °C for 5 min. Lysates containing 15 μg protein were subjected to SDS-polyacrylamide gel electrophoresis, and the separated proteins were transferred to nitrocellulose membranes. The membranes were blocked with 2.5% skimmed milk and 0.25% bovine serum albumin in Tris-buffered saline (50 mM Tris-HCl and 150 mM NaCl, pH 7.4) containing 0.1% Tween 20 for 60 min at room temperature, and then incubated with appropriate primary antibodies overnight at 4 °C. The membranes were then incubated with an appropriate secondary antibody for 60 min at room temperature. Finally, the membranes were incubated with ImmunoStar LD (Wako). Specific protein bands were visualized using ChemiDoc (Bio-Rad, Hercules, CA, USA), and the data were analyzed using Image Lab software. For figure preparation, brightness and contrast adjustments were applied uniformly across the entire image and equally to all samples within the same blot.

For all Western blot experiments, membranes were cut prior to antibody hybridization to allow simultaneous probing with different antibodies. Original uncropped blot images, including membrane edges and all replicates, are provided in the Supplemental Information.

### 2.10 Collection of small extracellular vesicles (sEVs)

Exosomes were isolated by ultracentrifugation. Prior to exosome collection, cells were cultured in exosome-free medium (DMEM supplemented with 10% exosome-depleted FBS and 1% penicillin-streptomycin) for 48 h after reaching confluence. The culture supernatant was sequentially centrifuged at 200 × g and 4 °C for 10 min, 2000 × g and 4 °C for 10 min, and 10,000 × g and 4 °C for 50 min to remove cells and debris. The resulting supernatant was passed through 0.22 μm filters (Millex-GV, PVDF, Merck Millipore). Exosomes were then pelleted by ultracentrifugation at 100,000 × g for 70 min using a Himac P70AT fixed-angle rotor. The exosome pellets were resuspended either in 30 μL of sample buffer for immunoblotting or in particle-free, 0.2 μm-filtered PBS for nanoparticle tracking analysis (NanoSight).

### 2.11 Nanoparticle tracking analysis (NTA)

The diameter and concentration of exosomes were measured using a NanoSight NS500 instrument equipped with a 532 nm laser and NTA 3.1 software. Prior to analysis, exosome samples were diluted in particle-free PBS to achieve the optimal measurement range (∼100 particles per field of view). Measurements were performed according to the manufacturer's instructions. NTA data were exported to Excel, and particle concentrations within each size range were calculated.

### 2.12 Reanalysis of publicly available human scRNA-seq data

Publicly available single-cell RNA-seq data from human SVF of sWAT and vWAT were obtained from the Gene Expression Omnibus (GEO) under accession number GSE129363 [5]. Raw count matrices in Matrix Market format, along with gene and cell barcode annotation files, were downloaded and analyzed in R (version 4.5.0) using the Seurat package (version 5.4.0). A Seurat object was created using the CreateSeuratObject function with thresholds of min.cells = 3 and min.features = 200. Cell-level annotations provided in the original dataset were imported and added to the Seurat object as metadata.

The proportion of mitochondrial transcripts was calculated using PercentageFeatureSet with the pattern “^∧^MT-”. Quality control metrics, including the number of detected genes, total UMI counts, and mitochondrial gene percentage, were visualized using violin plots. Cells expressing fewer than 300 genes, more than 6000 genes, or with mitochondrial gene content exceeding 15% were excluded from downstream analyses. The filtered data were normalized using NormalizeData, and highly variable genes were identified using FindVariableFeatures with 3000 features. Data scaling was performed using ScaleData, followed by principal component analysis (PCA) using RunPCA with 50 principal components. The number of principal components used for downstream analyses was determined based on elbow plots.

A shared nearest-neighbor graph was constructed using FindNeighbors with the first 30 principal components, followed by graph-based clustering using FindClusters at a resolution of 0.5. Uniform Manifold Approximation and Projection (UMAP) was performed using RunUMAP with the same principal components for visualization. Cluster identities and tissue-of-origin (sWAT vs. vWAT) distributions were visualized using UMAP embeddings.

To identify major stromal and immune lineages within the SVF, module scores were calculated using AddModuleScore based on established marker gene sets. The APC signature consisted of PDGFRA, CD34, DCN, LUM, COL1A1, COL3A1, DPT, PI16, CXCL14, CFD, APOD, and WISP2 [3,13,14]. The endothelial cell signature included PECAM1, VWF, KDR, ESAM, and CLDN5 [15]. The pericyte signature comprised RGS5, MCAM, CSPG4, PDGFRB, ACTA2, and TAGLN [15]. The immune cell signature included PTPRC, LYZ, LST1, C1QA, NKG7, CD3D, and MS4A1 [16]. Module score distributions were compared across clusters using violin plots.

Clusters enriched for the APC module score were subsetted for higher-resolution analysis. The subsetted cells were reprocessed independently, including normalization, identification of 3000 variable features, scaling based on variable genes, and PCA using 50 principal components. Graph-based clustering was performed using FindNeighbors with the first 25 principal components, followed by FindClusters at a resolution of 0.6, and UMAP visualization using the same principal components. Expression of candidate genes, including *LGALS9*, CD44, and *CD63*, was assessed using violin plots, with comparisons stratified by tissue of origin.

### 2.13 Iron-induced cell death measurements

CD63-OE 3T3-L1 cells were treated with 4 mM FAC in maintenance medium for 24 h. Cells were then detached using Accutase™ and centrifuged at 300 × g and 4 °C for 10 min. The resulting cell pellets were resuspended in FACS buffer. Cells were stained with propidium iodide (PI) and incubated in the dark for 30 min at 4 °C. After incubation, cells were washed, centrifuged, and resuspended in FACS buffer, followed by filtration through a 70 μm cell strainer. PI-stained cells were analyzed using a BD FACSMelody™ flow cytometer.

## 3 Statistics

All quantitative data are presented as mean ± SD. Statistical analyses were performed using GraphPad Prism. Statistical tests were applied to key quantitative data as indicated in the figure legends. Exact sample sizes (*n*) and definitions of *n* (number of animals, number of independent cell cultures, or number of independent experiments) are provided in the corresponding figure legends.

Statistical analyses were performed using independent replicates obtained from separate experiments conducted on different days. Technical replicates were not used for hypothesis testing. No statistical methods were used to predetermine sample size. No data were excluded from the analyses. No randomization or blinding was applied unless otherwise stated.

For comparisons between two groups, two-tailed Student's *t*-tests were used. For experiments involving two independent variables, two-way analysis of variance (ANOVA) was performed with genotype (mock vs CD63-OE) and treatment (FAC– vs FAC+) as factors. Statistical significance was defined as *P* < 0.05.

## 4 Results

### 4.1 Investigation of differences in cell surface protein expression between s-APCs and v-APCs

**Fig. 1:**
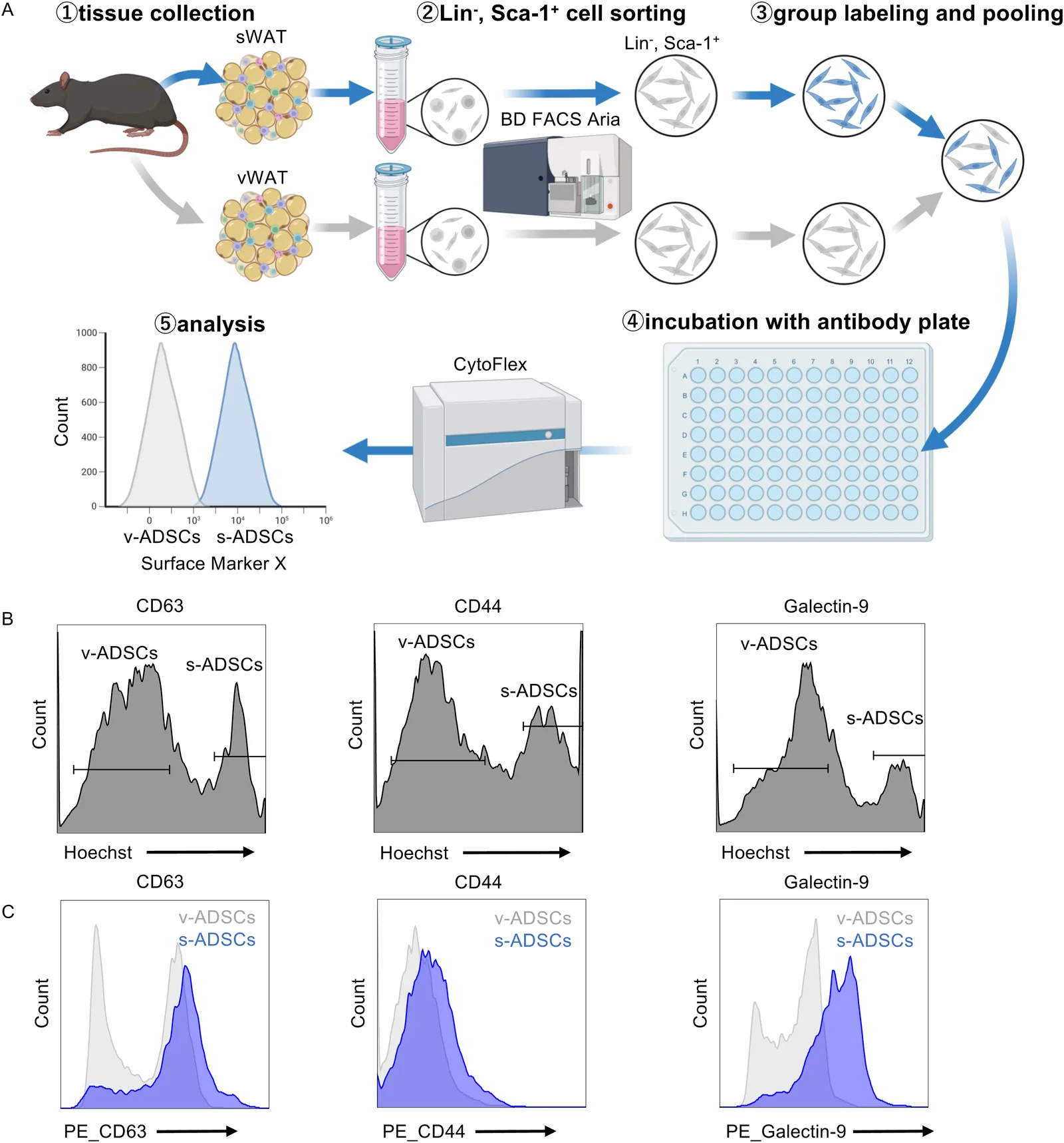
Schematic representation of the experimental procedure and the results of initial screening (A) After euthanasia, subcutaneous white adipose tissue (sWAT) and visceral white adipose tissue (vWAT) were minced and digested with collagenase type II. The digested tissues were then filtered and collected separately. Lineage-negative (Lin^–^)/Sca-1-positive (Sca-1^+^) cells were isolated using a flow cytometer (BD FACS Aria III). Lin^–^ Sca-1^+^ cells from sWAT were stained with Hoechst dye and subsequently mixed with Lin^–^ Sca-1^+^ cells from vWAT. The mixed Lin^–^ Sca-1^+^ cell population was incubated on an antibody-coated 96-well plate containing PE-conjugated antibodies against diverse cell-surface antigens, and stained cells were analyzed by flow cytometry (CytoFLEX). A complete list of screened antigens is provided in Fig. S2. The image was created with Biorender.com. (B) Histogram showing Hoechst fluorescence to distinguish the origin of Lin^–^ Sca-1^+^ cells. Hoechst^+^ cells were defined as s-APCs, whereas Hoechst^–^ cells were v-APCs. (C) Representative histograms showing PE fluorescence intensity of three candidate surface markers identified in the screening: CD63, galectin-9, and CD44. Blue histograms represent s-APCs Lin^–^ Sca-1^+^ cells, and gray histograms represent v-APCs. Gates were determined by Hoechst fluorescence peak separation and were slightly adjusted between experiments to minimize overlap between the two populations.

The cellular characteristics of subcutaneous and visceral APCs were compared by conducting a surface protein screening of APCs isolated from sWAT and vWAT fat depots. An overview of the experimental design is presented in Fig. 1A and summarized below. Hematopoietic lineage negative (Lin^–^)/Sca-1^+^ stromal cells were isolated from sWAT and vWAT according to the gating strategy depicted in Fig. S1. sWAT-derived Lin^–^ Sca-1^+^ cells were labeled with Hoechst dye, pooled with unlabeled vWAT-derived cells, and incubated on plates coated with PE-conjugated antibodies targeting cell-surface proteins. Surface protein screening was performed using a panel of PE-conjugated antibodies recognizing diverse cell-surface antigens. Representative candidate markers identified during screening are summarized in Table 1, and the complete screening dataset is provided in Fig. S2. A plate-based flow cytometer platform enabled discrimination of s-APCs (Hoechst-high) and v-APCs (Hoechst-low), allowing direct comparison of PE fluorescence intensity as a measure of surface protein expression. Among these, CD44, CD63, and galectin-9 emerged as prominent candidates that contribute to the functional divergence between s-APCs and v-APCs (Fig. 1B and C). Subsequent analyses focused on these proteins to define their depot-specific expression patterns and functional relevance.

**Table 1:** Representative surface proteins with notable differences.

Higher in s-APCs	Higher in v-APCs
ESAM	DLL1
PDC-TREM	CD107b (Mac-3)
CD301a (MGL1)	CD51
CD278 (ICOS)	JAML
CD44	CD210 (IL-10 R)
CD105	CD61
Galectin-9	CD144 (VE-cadherin)
CD200 (OX2)	CD117 (c-kit)
CD140a	Notch 2
CD71	CD81
CD63	Notch 3
CD146	TLT-2
	Ly-51
	CD276 (B7-H3)

Abbreviations.

s-APCs, subcutaneous adipose progenitor cells; v-APCs, visceral adipose progenitor cells.

ESAM, endothelial cell-selective adhesion molecule; DLL1, delta-like ligand 1.

PDC-TREM, plasmacytoid dendritic cell–triggering receptor expressed on myeloid cells.

MGL1, macrophage galactose-type lectin 1; ICOS, inducible T-cell co-stimulator.

JAML, junctional adhesion molecule-like protein; IL-10R, interleukin-10 receptor.

VE-cadherin, vascular endothelial cadherin; c-Kit, KIT proto-oncogene receptor tyrosine kinase.

TLT-2, triggering receptor expressed on myeloid cells-like transcript 2.

**Table 2:** Primers used for this study.

Application	Target	Type	Sequence
cloning	*Lgals9*	F	5′-GGG GGA ATT CGC CAC CAT GGC TCT CTT CAG TGC CCA GTC TCC-3′
		R	5′-GGG GCT CGA GCT ATG TCT GCA CGT GGG TCA GCT GG-3′
cloning	*CD44*	F	5′-GGG GGA ATT CGC CAC CAT GGA CAA GTT TTG GTG GCA CAC AGC T-3′
		R	5′-GGG GCT CGA GCT ACA CCC CAA TCT TCA TGT CCA CAC TCT GC-3′
cloning	*CD63*	F	5′-GGG GGA ATT CGC CAC CAT GGC GGT GGA AGG AGG AAT GAA GTG-3′
		R	5′-GGG GCT CGA GCT ACA TTA CTT CAT AGC CAC TTC GAA TAC TCT TCA-3′
qPCR	*Lgals9*	F	5′-TGC CCA GTC TCC ATA CAT TAA CC-3′
		R	5′-AAA CTC TTG GTA GTC CCC TGG AG-3′
qPCR	*Cd44*	F	5′-CAT CCC AAC GCT ATC TGT GC-3′
		R	5′-CAT CGA AGG AAT TGG GTA GGT C-3′
qPCR	*Cd63*	F	5′-CTC TTA TCA TGC TTG TGG AGG TG-3′
		R	5′-TCT GCA TCT GCT GCT GGA AG-3′
qPCR	*Pparg2*	F	5′-TGT TGA CCC AGA GCA TGG TG-3′
		R	5′-CAC AGA GCT GAT TCC GAA GTT G-3′
qPCR	*Cebpa*	F	5′-GCC TTC AAC GAC GAG TTC C-3′
		R	5′-CCC GGG TAG TCA AAG TCA CC-3′
qPCR	*Adipoq*	F	5′-TGC CGA AGA TGA CGT TAC TAC AAC-3′
		R	5′-CTT CAG CTC CTG TCA TTC CAA C-3′
qPCR	*Rps18*	F	5′-TGC GAG TAC TCA ACA CCA ACA T-3′
		R	5′-CTT TCC TCA ACA CCA CAT GAG C-3′

### 4.2 Validation and characterization of galectin-9 and CD44 as functional surface proteins in s-APCs

**Fig. 2:**
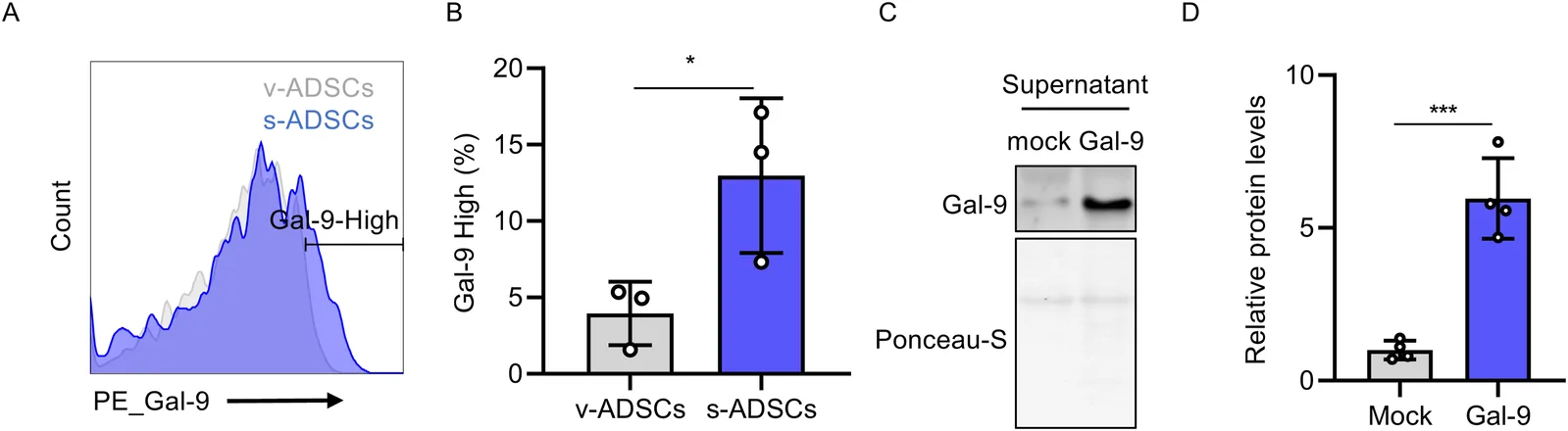
Validation of galectin-9 (Gal-9) expression in APCs and analysis in Gal-9-OE 3T3-L1 cells (A) Flow cytometry histograms showing Gal-9 expression in s-APCs (blue) and v-APCs (gray) stained with the PE-conjugated anti-galectin-9 antibody. Quantification is shown in (B) (*n* = 3, *p* = 0.046). (C) Western blotting and (D) quantitative analysis of Gal-9 in culture supernatants of mock and Gal-9-OE 3T3-L1 cells. (*p* = 0.0003). Data are expressed as means ± SD from independent biological replicates obtained from separate experiments and analyzed by the Student's *t*-test; *P < 0.05, ***P < 0.001.

Validation of the surface protein screening results began with examining galectin-9 expression by flow cytometry (Fig. 2A and B). Although galectin-9 levels differed between s-APCs and v-APCs at the cell surface, *Lgals9* (galectin-9) mRNA expression showed no significant difference between whole sWAT and vWAT (Fig. S3A). To determine whether this discrepancy reflects cell type-specific expression differences, we reanalyzed publicly available single-cell RNA-seq data from human stromal vascular fraction (SVF) cells derived from sWAT and vWAT (GSE129363) [5] (Fig. S4A–D).

Unsupervised clustering of SVF cells identified multiple distinct cell populations, which were annotated based on the expression of established lineage-specific marker genes (Fig. S4A and B). Among these clusters, clusters 2, 4, 5, 9, and 10 exhibited characteristic expression patterns of APC markers and were therefore classified as APC populations (Fig. S4C). Comparative analysis of *LGALS9* expression between APCs in sWAT and vWAT revealed that *LGALS9* transcripts were not distinctly detected in either population, and no significant difference in *LGALS9* expression was observed at the transcriptomic level (Fig. S4D), despite the observed depot-specific difference in surface galectin-9 abundance.

The functional role of galectin-9 in APCs was examined using galectin-9 overexpressing (OE) 3T3-L1 (Gal-9-OE) preadipocytes as a model for s-APCs to model the elevated galectin-9 state observed in s-APCs. Successful overexpression was confirmed by RT-PCR (Fig. S3B). Potential confounding interactions with serum-derived transferrin, which interacts with galectins in a glycan-dependent manner [17], were minimized by culturing Gal-9-OE cells in standard maintenance medium until 24 h before analysis, followed by replacement with FBS-free DMEM. Flow cytometry confirmed elevated surface galectin-9 expression in Gal-9-OE cells (Fig. S3C). In line with its secretory nature, galectin-9 release into the culture supernatant was assessed by western blotting and found to be increased in Gal-9-OE cells compared with Mock-control cells (Fig. 2C and D).

**Fig. 3:**
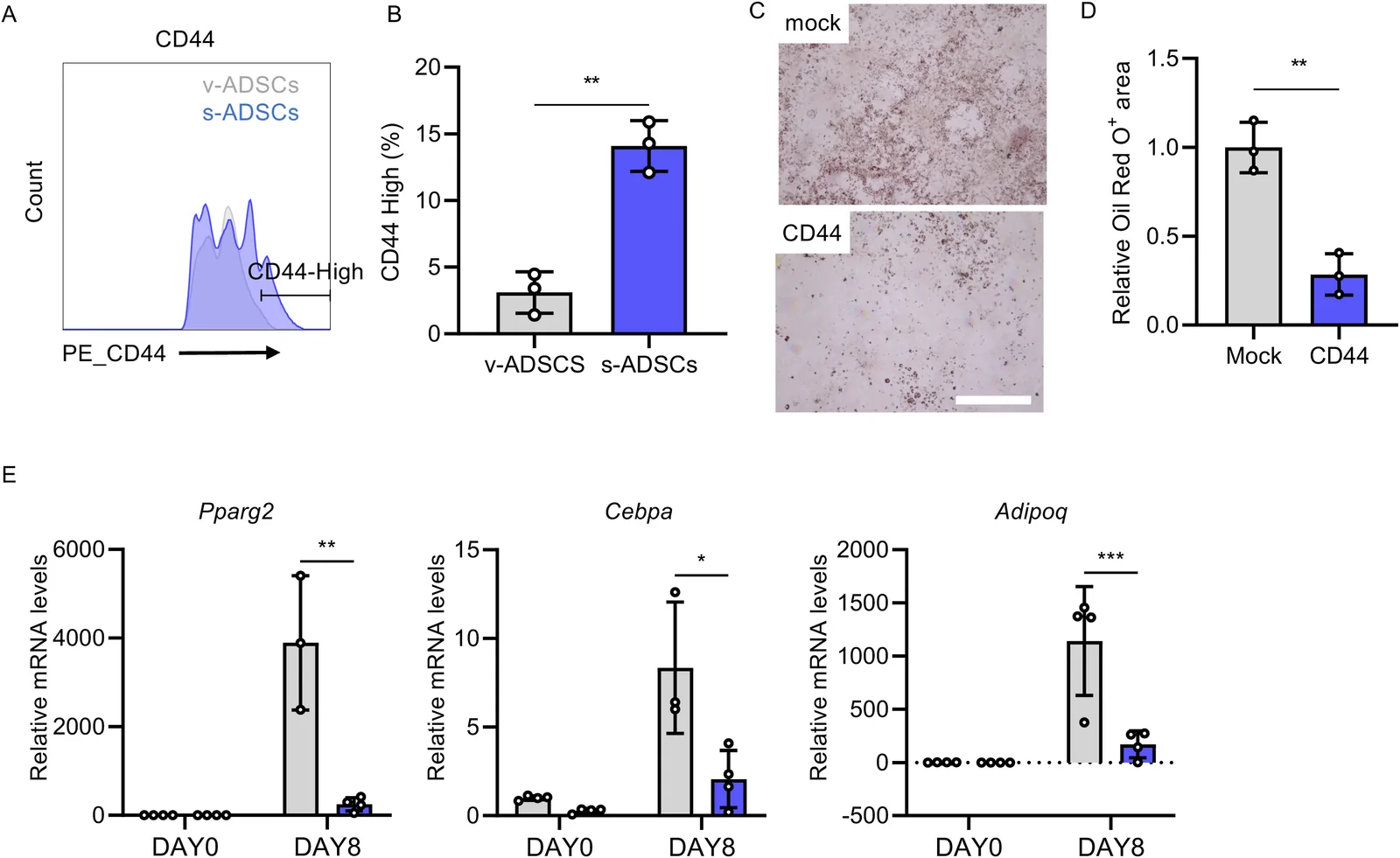
Validation of CD44 expression in APCs and analysis in CD44-OE 3T3-L1 cells (A) Flow cytometry histograms showing CD44 expression in s-APCs (blue) and v-APCs (gray) stained with the PE-conjugated anti-CD44 antibody. Quantification is shown in (B) (*n* = 3, *p* = 0.001). (C) Representative microscopic images of Oil Red O-stained mock and CD44-OE cells at 8 days post-adipogenic differentiation induction. Quantification is shown in (D) (*n* = 3, *p* = 0.002). (E) Relative mRNA levels of adipogenic regulators and adipocyte markers, including *Pparg* isoform 2 (*Pparg2*), *Cebpa*, and *Adipoq*, in mock and CD44-OE 3T3-L1 cells at days 0 and 8 of adipogenic differentiation. Gene expression levels were normalized to *Rps18* and expressed relative to mock cells at day 0. Statistical comparisons were performed between mock and CD44-OE cells at day 8 using the Student's *t*-test. (*n* = 3–4) Data are expressed as means ± SD from independent biological replicates obtained from separate experiments and analyzed by the Student's *t*-test; *P < 0.05, **P < 0.01, ***P < 0.001. Scale bar, 400 μm.

CD44, another surface protein enriched in s-APCs, was subsequently examined. Although *Cd4*4 mRNA levels were comparable between depots in whole WAT (Fig. S5A), CD44 surface expression was significantly higher in s-APCs than in v-APCs (Fig. 3A and B). Consistent with this observation, *CD4*4 mRNA levels were also elevated in s-APCs (Fig. S4D). Functional assessment using CD44-OE 3T3-L1 cells revealed impaired adipogenic differentiation, as indicated by reduced lipid accumulation compared with controls (Fig. 3C, D, S5B, C). Consistent with this observation, the expression of adipogenic regulators and adipocyte markers, including *Pparg* (isoform 2), *Cebpa*, and *Adipoq*, was significantly reduced in differentiated CD44-OE cells compared with mock controls (Fig. 3E). These findings suggest that elevated CD44 levels promote maintenance of a progenitor-like state by suppressing differentiation. Collectively, elevated galectin-9 and CD44 expression in s-APCs appears to support an anti-inflammatory, undifferentiated phenotype.

### 4.3 Elevated CD63 expression is associated with increased exosome release

**Fig. 4:**
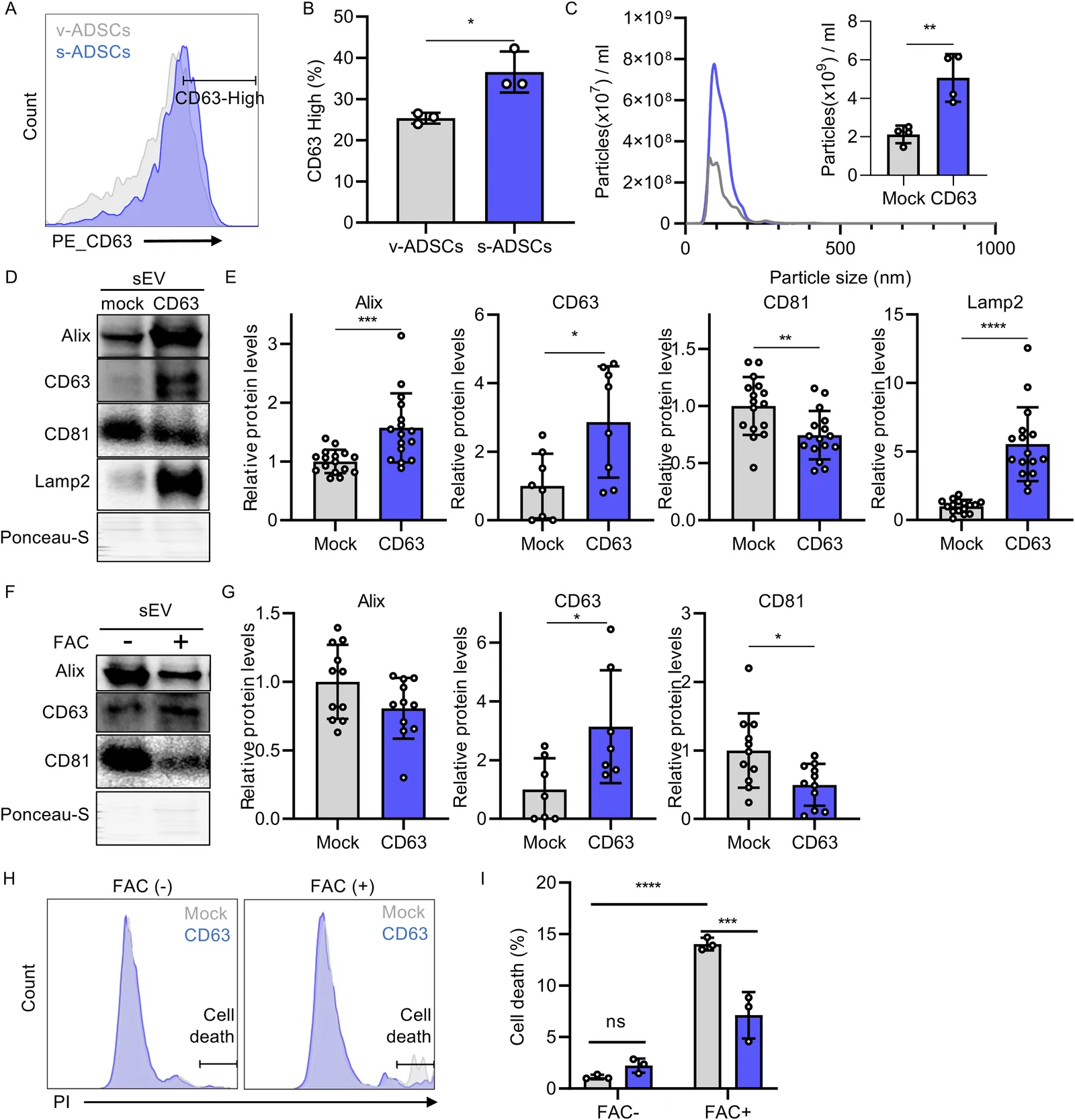
Validation of CD63 expression in APCs and analysis in the CD63-OE 3T3-L1 cell line (A) Flow cytometry histograms showing CD63 expression in s-APCs and v-APCs stained with the PE-conjugated anti-CD63 antibody. Quantification is shown in (B) (*n* = 3, *p* = 0.01). (C) Distribution curve and box plot of extracellular vesicle size in CD63-OE 3T3-L1 cells (*p* = 0.004). (D) Western blotting and (E) quantitative analysis of Alix (*p* = 0.005), CD63 (*p* = 0.01), CD81 (*p* = 0.02), and Lamp2 (*p* = 0.00001) in sEV released by CD63-OE 3T3-L1 cells. (F) Western blotting and (G) quantitative analysis of Alix, CD63 (*p* = 0.01), and CD81 (*p* = 0.01) in sEV released by Ferric ammonium citrate (FAC)-treated 3T3-L1 cells. (H) Representative flow cytometry histograms and quantification (I) of propidium iodide (PI) staining in mock and CD63-OE 3T3-L1 cells treated with FAC (4 mM) for 24 h. Data are expressed as means ± SD from independent biological replicates obtained from separate experiments and analyzed by the Student's *t*-test (A–G) or two-way ANOVA with genotype (mock vs. CD63-OE) and treatment (FAC^–^ vs. FAC^+^) as factors (H, I); *P < 0.05, **P < 0.01, ***P < 0.001.

CD63 is a well-established tetraspanin enriched on the surface of exosomes and implicated in exosome biogenesis and intracellular trafficking [18,19]. We first confirmed the differential surface expression of CD63 between s-APCs and v-APCs by flow cytometry (Fig. 4A and B).

In contrast to the pronounced difference observed at the cell surface, CD63 mRNA levels did not differ significantly between sWAT and vWAT in bulk adipose tissue RNA (Fig. S6A). Reanalysis of publicly available scRNA-seq data also revealed no significant difference in CD63 transcript levels between sWAT- and vWAT-derived APCs (Fig. S4D). These observations indicate that elevated surface CD63 expression in s-APCs is regulated post-transcriptionally. Given the established role of CD63 in exosome biology, we next examined whether increased CD63 expression correlates with altered exosome release and composition. CD63-overexpressing 3T3-L1 preadipocytes (CD63-OE) were generated as a surrogate model to mimic CD63^high^ s-APCs. CD63-OE cells released significantly more exosomes than control cells, whereas no differences were observed in exosome size distribution (Fig. 4C and S6B, C, D, E). Exosomes derived from CD63-OE cells also showed increased levels of the exosomal markers ALG-2–interacting protein X (Alix), CD63, and lysosome-associated membrane protein 2 (LAMP2/CD107b), accompanied by reduced CD81 expression (Fig. 4D and E). This result suggests a shift in exosomal surface protein composition; however, the functional implications of altered CD81 levels remain to be clarified. A similar pattern was observed in the surface proteomic profiles of s-APCs (Table 1), supporting the notion that elevated CD63 expression is linked to altered exosome output and composition. Collectively, these results indicate that CD63 not only serves as a classical exosome marker but also contributes to altered exosome release and composition.

Previous studies have reported that vWAT is more susceptible to iron-dependent inflammation than sWAT [20] and that human fibroblasts release iron ions via CD63^high^ exosomes [21]. Given the higher CD63 expression in s-APCs and its reported role in iron export via exosomes, we hypothesized that elevated CD63 expression in s-APCs may influence exosome-mediated iron handling. Iron stress was induced in 3T3-L1 cells using ferric ammonium citrate (FAC), followed by analysis of exosome-associated proteins. Western blot analysis revealed that iron stress increased CD63 levels and decreased CD81 levels in exosomes (Fig. 4F, G), a pattern reminiscent of the surface marker profile observed in our initial screening (Fig. S2; Table 1). Furthermore, CD63-OE cells were more resistant to iron-induced cell death than control cells (Fig. 4H and I). These findings indicate that high CD63 expression not only marks s-APCs but also enhances their exosome-mediated adaptation to iron-induced stress, thereby contributing to stress resilience.

## 5 Discussion

In this study, we show that s-APCs exhibit distinct surface protein profiles associated with inflammatory responses and adipogenic capacity, in a depot-dependent manner. Elevated surface expression of CD63 and galectin-9 in s-APCs correlated with a less inflammatory microenvironment, whereas CD44 expression levels modulated APC adipogenic potential in context- and dose-dependent manners. These results highlight surface protein heterogeneity as a regulatory layer linking APC identity to adipose tissue remodeling and depot-specific traits.

Our findings suggest that s-APCs share a coordinated surface protein signature that may confer resistance to inflammation. A central feature of this program is the high surface expression of CD63. CD63^high^ APCs released increased amounts of exosomes characterized by a CD63^high^/Lamp2^high^/CD81^low^ composition and exhibited enhanced resistance to iron-induced cell death. This exosomal profile closely resembles that reported in human fibroblasts, in which CD63^high^/CD81^low^ extracellular vesicles mediate iron export and protect cells from iron-induced toxicity [21].

Consistent with this model, an iron overload increased the release of CD63^high^/CD81^low^ exosomes in our system, a profile that resembles the higher CD63 and lower CD81 surface protein expression observed in s-APCs compared with v-APCs during the initial screening. Given the reported susceptibility of vWAT to iron-dependent inflammation relative to sWAT [20], enhanced exosome-mediated iron handling in s-APCs may contribute to the lower inflammatory state of sWAT. In addition, our surface protein screening identified higher CD71 expression on s-APCs. CD71 mediates transferrin-dependent iron uptake and plays a central role in cellular iron metabolism [[22], [23], [24], [25]]. Together with CD63-dependent exosomal iron export, this observation supports an iron-handling program in s-APCs that may afford resistance to iron-driven inflammatory stress.

Galectin-9 also emerged as a prominent surface molecule enriched in s-APCs. APCs with high surface galectin-9 expression secreted increased levels of soluble galectin-9. Galectin-9 exerts immunosuppressive effects, including the induction of T-cell apoptosis via TIM-3 signaling and the modulation of macrophage polarization toward anti-inflammatory phenotypes [[26], [27], [28]]. Notably, galectin-9 can be packaged into CD63-positive extracellular vesicles [29], suggesting a mechanistic link between elevated CD63 expression and enhanced galectin-9 secretion. Collectively, these findings suggest that s-APCs actively shape a low-inflammatory microenvironment by coordinating the regulation of exosome biogenesis, iron handling, and the release of immunomodulatory factors.

Depot-specific surface protein expression patterns also influenced adipose tissue expansion. CD44 surface expression differed between subcutaneous and visceral APCs, and elevated CD44 expression was associated with impaired adipogenic differentiation *in vitro*. These findings align with previous reports showing that CD44 can promote or inhibit adipogenesis depending on the experimental context [[30], [31], [32]].

From this perspective, CD44 differences may contribute to depot-specific expansion patterns: sWAT favors hyperplastic growth, whereas vWAT expands through hypertrophy of existing adipocytes. These progenitor behaviors may underlie depot-specific susceptibility to metabolic stress. For example, pathogenic factors such as ZNF746 (also known as PARIS) preferentially accumulate in vWAT, impairing mitochondrial biogenesis and promoting dysfunctional remodeling [33]. Intrinsic differences in APC identity, inflammatory tone, and differentiation capacity may shape such depot-specific accumulation patterns. Although the precise molecular mechanisms remain unresolved, variation in CD44 expression may bias APC responsiveness to adipogenic cues, promoting sWAT progenitor recruitment and adipogenesis while limiting these processes in vWAT.

Notably, galectin-9 has been identified as a ligand for CD44 [34], and galectin-9-dependent CD44 signaling regulates cell number and size expansion in hepatocytes [35]. Differences in galectin-9 and CD44 expression between sWAT- and vWAT-derived APCs may therefore contribute to depot-specific modes of WAT expansion. Because both galectin-9 and CD44 were preferentially elevated in s-APCs, their interaction may be more prominent in sWAT than in vWAT, potentially influencing progenitor behavior and tissue remodeling in a depot-dependent manner. However, the mechanistic role of the galectin-9/CD44 signaling axis in APCs has not yet been directly established through functional characterization. Importantly, galectin-9 and CD44 are unlikely to be sole determinants of adipose tissue expansion, underscoring the importance of quantitative signal integration and cellular context.

Although recent single-cell transcriptomic analyses have advanced our understanding of APC heterogeneity [3,5], transcript abundance does not necessarily predict protein localization, surface availability, or functional activity. In particular, galectin-9 surface expression differed between s-APCs and v-APCs despite the absence of clear depot-specific differences in *Lgals9* transcript abundance. This discrepancy suggests that transcript levels alone may not fully account for surface galectin-9 abundance and may reflect differences in protein trafficking, membrane localization, secretion efficiency, or subtle variations in APC subtype composition. In this study, CD63 and galectin-9 did not show distinct depot-specific differences at the transcript level in publicly available scRNA-seq datasets, despite apparent differences in surface expression and functional outcomes. In contrast, CD44 displayed consistent depot-specific differences at both transcript and surface protein levels. Conversely, CD200, previously reported as a v-APC-enriched surface protein [36], displayed an inverted expression pattern in our assay, with higher surface expression in sWAT-derived cells. This divergence highlights how experimental conditions and detection strategies can shape surface protein profiles. Given WAT and APC plasticity [6], direct surface protein expression analysis provides a complementary, functionally informative layer that cannot be readily inferred from transcriptomic data alone.

Previous lineage-tracing studies have shown that vWAT progenitors primarily derive from WT1-expressing mesothelial cells of the lateral plate mesoderm, whereas sWAT does not arise from this WT1^+^ lineage [37]. Consistent with this concept, transplantation experiments show that APC transcriptional programs are primarily determined by tissue of origin rather than the recipient microenvironment [5]. In this context, the surface protein differences identified herein likely reflect intrinsic regulatory programs of s-APCs that persist independently of environmental cues.

## 6 Limitations and future directions

Several limitations of the present study should be acknowledged. Although APC populations are increasingly recognized as heterogeneous, the present study focused on identifying depot-specific surface protein signatures in the overall Lin^−^ Sca-1^+^ APC population rather than subdividing individual APC subtypes. In addition, although the Lin^−^ Sca-1^+^ flow-cytometric gating strategy enriches adipogenic progenitor cells, it may not completely exclude contributions from mesothelial-like cells and endothelial progenitor populations, particularly in vWAT. Therefore, their contribution to the observed depot-specific surface protein signatures cannot be entirely excluded. Future studies using additional lineage markers and higher-resolution approaches will be necessary to determine whether the identified differences reflect intrinsic molecular characteristics or differences in cellular composition between sWAT and vWAT. Furthermore, proteomics-based approaches, particularly those that enable APC-specific surface protein profiling, will be valuable to further validate depot-specific molecular signatures. Future studies integrating APC-specific proteomic and transcriptomic analyses, further stratifying APC subtypes, and directly characterizing the galectin-9/CD44 signaling axis will be important to define the molecular basis of depot-specific APC function. Validation using human adipose progenitor models, such as SGBS cells, will also be important to assess the translational relevance of the identified depot-specific surface protein signatures.

Collectively, our findings highlight surface protein identity as a key intrinsic property governing depot-specific behavior of adipose progenitor cells. By shaping susceptibility to inflammatory stress, differentiation potential, and responsiveness to environmental cues, these surface signatures provide a mechanistic framework for understanding how regional adipose depots differentially contribute to metabolic disease risk.

## Ethics declaration

This study was conducted in accordance with the ARRIVE (Animal Research: Reporting of In Vivo Experiments) guidelines. This study was approved by the Ethics Review Committee for Animal Experimentation at Tokyo University of Science. (Approval No. Y20043, Y21043, and Y22037)

## Resource availability

### Lead contact

Further information and requests for resources and reagents should be directed to and will be fulfilled by the lead contact, Yuhei Mizunoe (ymizunoe@rs.tus.ac.jp).

### Materials availability

All unique/stable reagents generated in this study are available from the lead contact on completing a Materials Transfer Agreement.

### Data and code availability

This study reanalyzed publicly available single-cell RNA-seq data from GEO (accession GSE129363).

Processed data generated in this study are available from the lead contact upon reasonable request.

Original code used for single-cell RNA-seq reanalysis (R/Seurat) is available upon request.

## Author contributions

Yuhei Mizunoe (Y.M.), Yuka Nozaki, and Yoshikazu Higami supervised the study. Kazuki Hachiya (K.H.) and Y.M. developed the study concept. K.H. and Y.M. wrote the main manuscript. Y.M. revised the manuscript. K.H., Nobuo Yamasaki, Hiroto Fukai, and Yuki Uchida performed the experiments and data analysis. All authors reviewed the manuscript and agreed to its submission.

## Funding

This work was supported by 10.13039/501100001691Grants-in-Aid from the Ministry of Education, Culture, Sports, Science and Technology of Japan (to Y. Mizunoe: Scientific Research (C) 25K1492). This work was supported by 10.13039/501100020961JST SPRING (to K. Hachiya: JPMJSP2151).

## Declaration of competing interests

The authors declare no competing interests.

## Data Availability

Data will be made available on request.
