# Supplementary material for: Surface protein profiling reveals depot-specific features of subcutaneous and visceral adipose progenitor cells
Source: Biochem Biophys Rep. 2026 Aug 17;47:102750. doi: 10.1016/j.bbrep.2026.102750 (PMC13507801; doi:10.1016/j.bbrep.2026.102750)
Supplement: Supplementary file 2 [file mmc2.pdf]

Figure S1

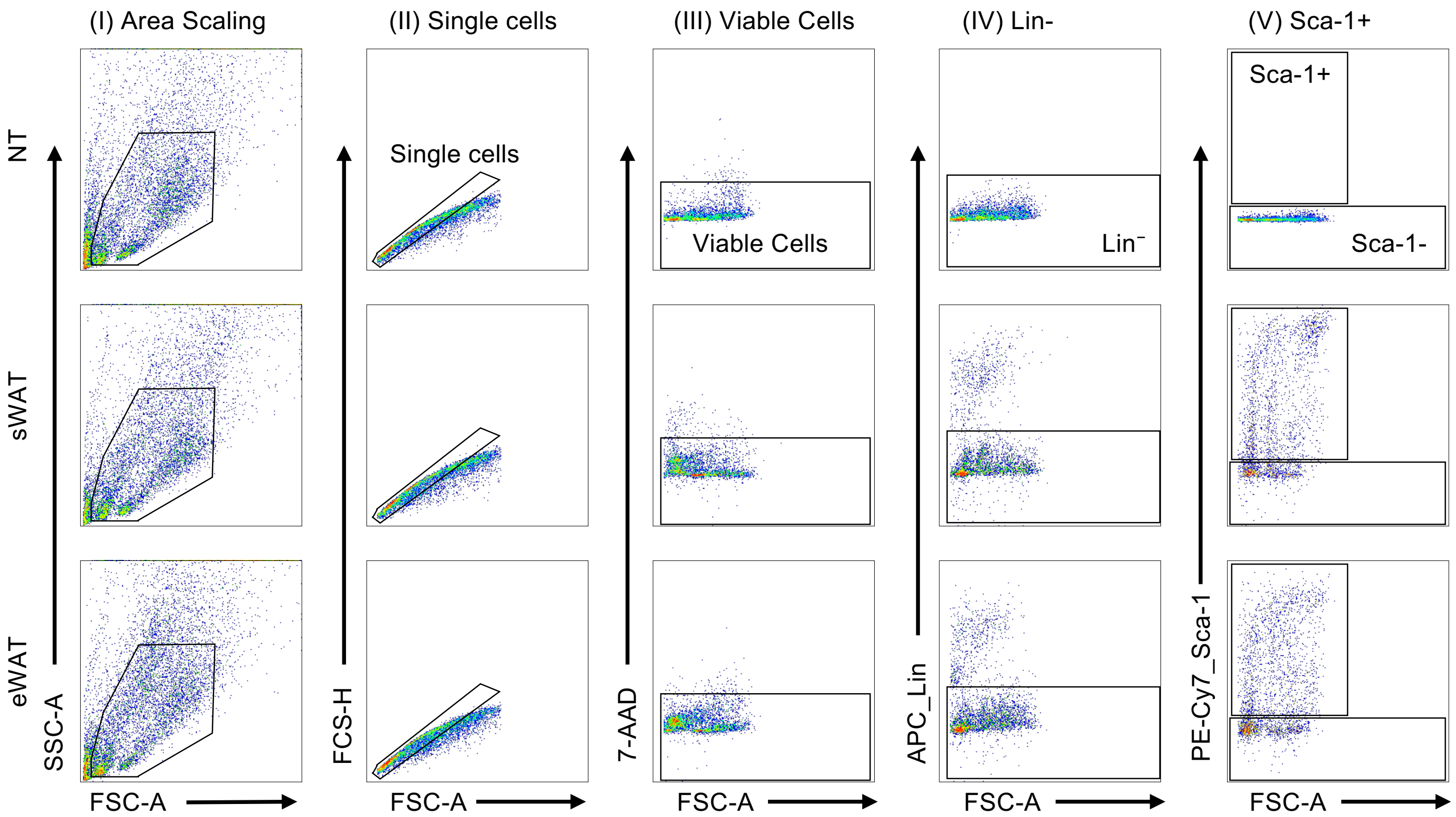

Figure S1. Gating strategy for the isolation of Lin⁻ Sca-1⁺ cells.
Cells were stained with 7-Aminoactinomycin D (7-AAD) and antibodies against TER119 (APC), CD45 (APC), CD31 (APC), and Sca-1 (PE-Cy7). Plots (I) to (V) illustrate the sequential gating strategy: (I) Initial gating was performed on FSC-A vs. SSC-A. (II) Single cells were identified using FSC-A vs. FSC-H. (III) Viable cells were selected by excluding 7-AAD–positive cells. (IV) Lineage-negative (Lin^-) cells were gated by excluding $TER119^+$, $CD45^+$, and $CD31^+$ populations. (V) $Sca\text{-}1^+$ cells were then selected from the Lin^- population.

Figure S2

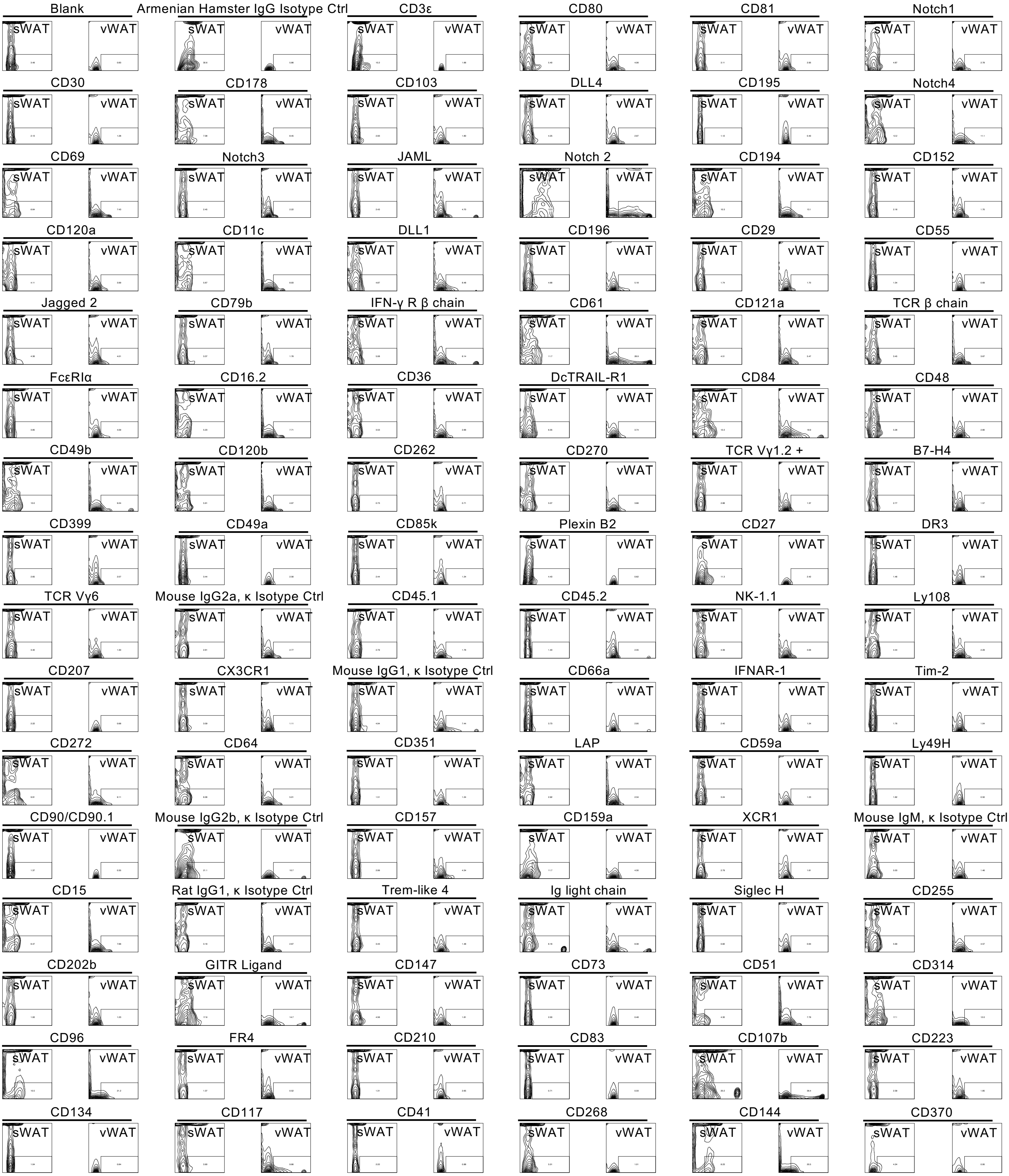
sWAT
vWAT
Blank
Armenian Hamster IgG Isotype Ctrl
CD3ε
CD80
CD81
Notch1
CD30
CD178
CD103
DLL4
CD195
Notch4
CD69
Notch3
JAML
Notch 2
CD194
CD152
CD120a
CD11c
DLL1
CD196
CD29
CD55
Jagged 2
CD79b
IFN-γ R β chain
CD61
CD121a
TCR β chain
FcεRIα
CD16.2
CD36
DcTRAIL-R1
CD84
CD48
CD49b
CD120b
CD262
CD270
TCR Vγ1.2 +
B7-H4
CD399
CD49a
CD85k
Plexin B2
CD27
DR3
TCR Vγ6
Mouse IgG2a, κ Isotype Ctrl
CD45.1
CD45.2
NK-1.1
Ly108
CD207
CX3CR1
Mouse IgG1, κ Isotype Ctrl
CD66a
IFNAR-1
Tim-2
CD272
CD64
CD351
LAP
CD59a
Ly49H
CD90/CD90.1
Mouse IgG2b, κ Isotype Ctrl
CD157
CD159a
XCR1
Mouse IgM, κ Isotype Ctrl
CD15
Rat IgG1, κ Isotype Ctrl
Trem-like 4
Ig light chain
Siglec H
CD255
CD202b
GITR Ligand
CD147
CD73
CD51
CD314
CD96
FR4
CD210
CD83
CD107b
CD223
CD134
CD117
CD41
CD268
CD144
CD370

Figure S2

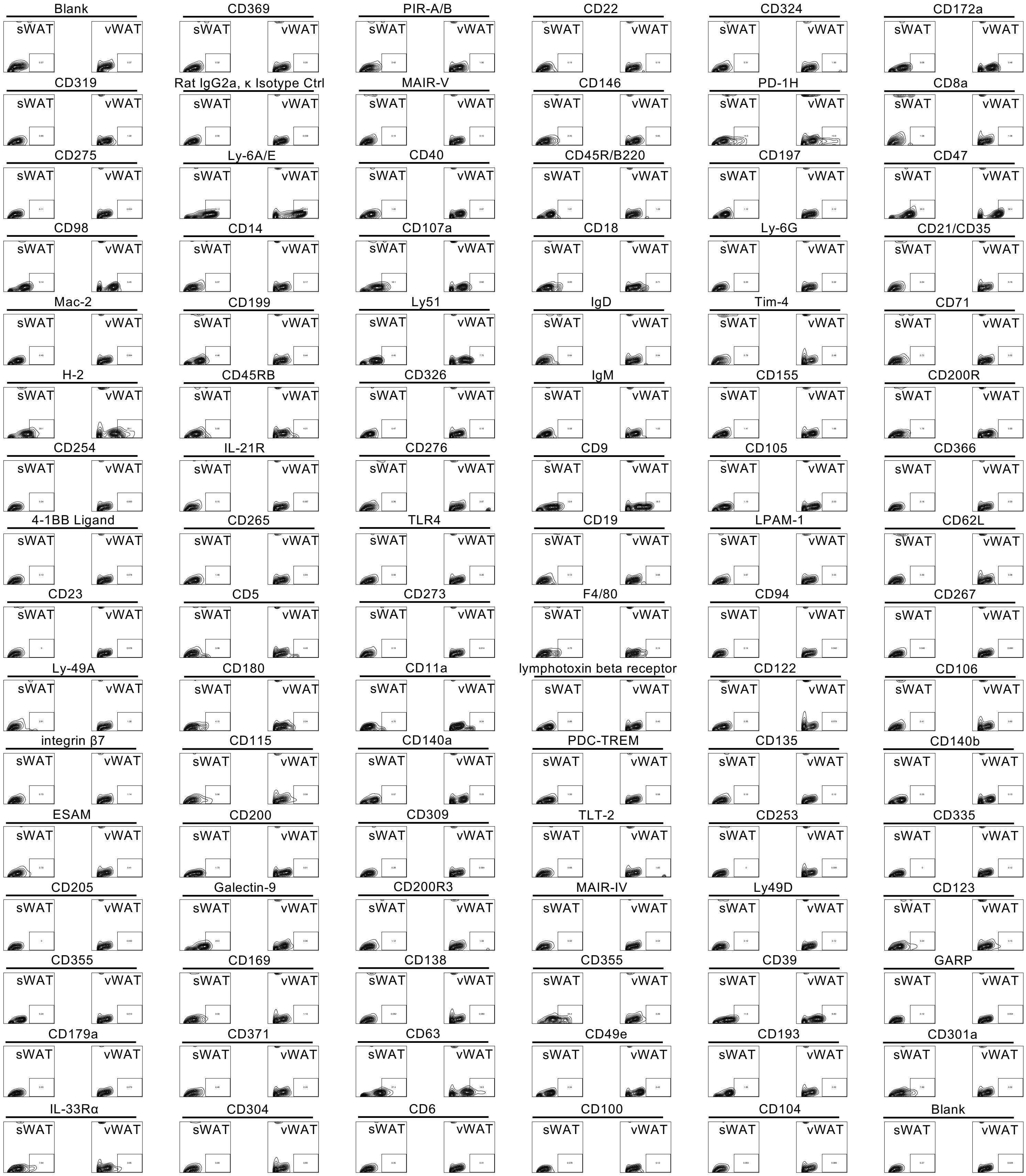
Blank
CD369
PIR-A/B
CD22
CD324
CD172a
CD319
Rat IgG2a, κ Isotype Ctrl
MAIR-V
CD146
PD-1H
CD8a
CD275
Ly-6A/E
CD40
CD45R/B220
CD197
CD47
CD98
CD14
CD107a
CD18
Ly-6G
CD21/CD35
Mac-2
CD199
Ly51
IgD
Tim-4
CD71
H-2
CD45RB
CD326
IgM
CD155
CD200R
CD254
IL-21R
CD276
CD9
CD105
CD366
4-1BB Ligand
CD265
TLR4
CD19
LPAM-1
CD62L
CD23
CD5
CD273
F4/80
CD94
CD267
Ly-49A
CD180
CD11a
lymphotoxin beta receptor
CD122
CD106
integrin β7
CD115
CD140a
PDC-TREM
CD135
CD140b
ESAM
CD200
CD309
TLT-2
CD253
CD335
CD205
Galectin-9
CD200R3
MAIR-IV
Ly49D
CD123
CD355
CD169
CD138
CD355
CD39
GARP
CD179a
CD371
CD63
CD49e
CD193
CD301a
IL-33Rα
CD304
CD6
CD100
CD104
Blank
sWAT
vWAT

Figure S2

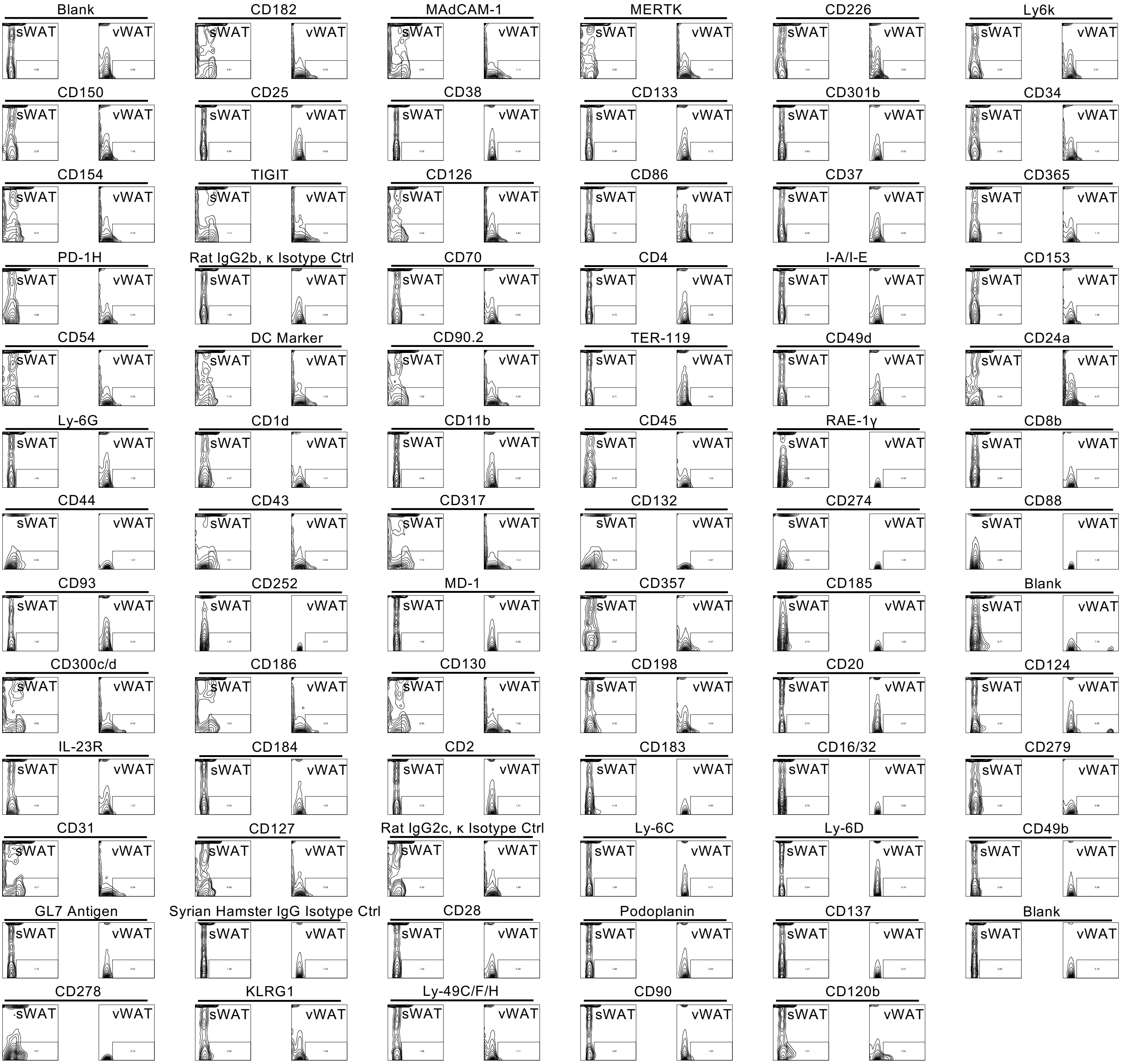

Figure S2. Full screening data.
Screening results for each indicated surface protein are shown as contour plots. The y-axis indicates SSC-A, and the x-axis indicates surface protein expression. In each panel, sWAT-derived cells are shown on the left and vWAT-derived cells on the right.

Figure S3

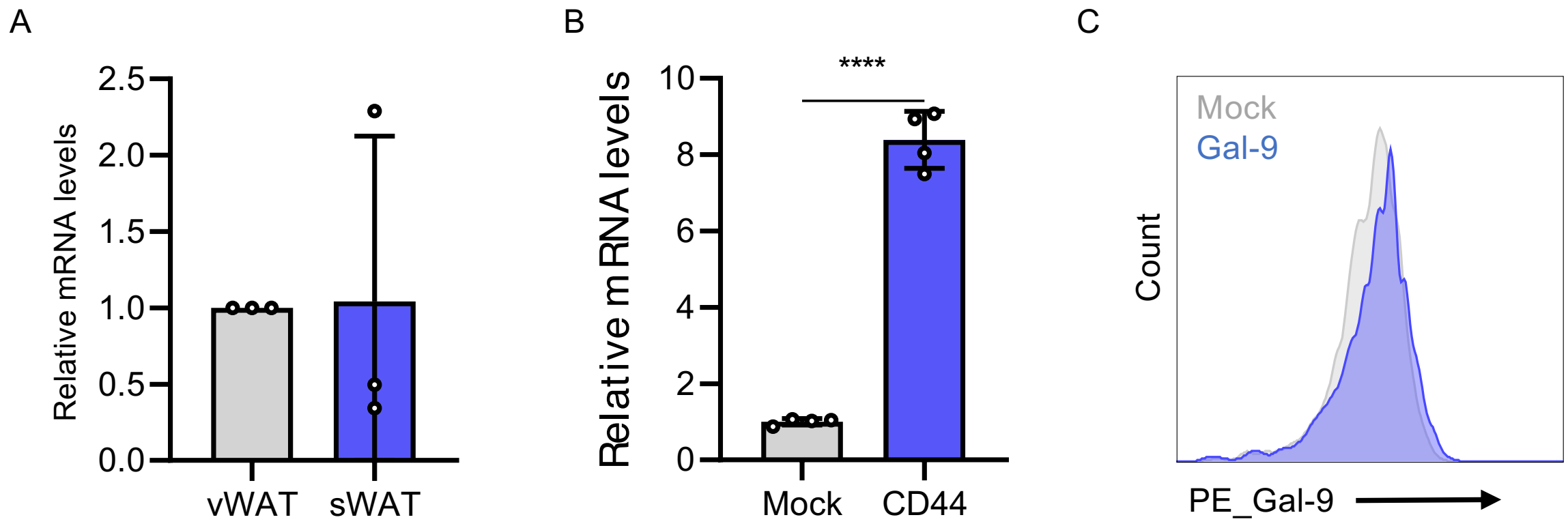

Figure S3. Additional data supporting Figure 2.
(A) Lgals9 (Galectin-9) mRNA levels in sWAT and vWAT. (B) Lgals9 (Galectin-9) mRNA levels in mock and Galectin-9 OE 3T3-L1 cells. (C) Flow cytometry histogram confirming Galectin-9 overexpression in 3T3-L1 cells using a PE-conjugated anti–Galectin-9 antibody ($P = 0.000001$). Data are expressed as the mean ± SD and were analyzed by Student's t-test; $^{***}P < 0.001$.

Figure S4

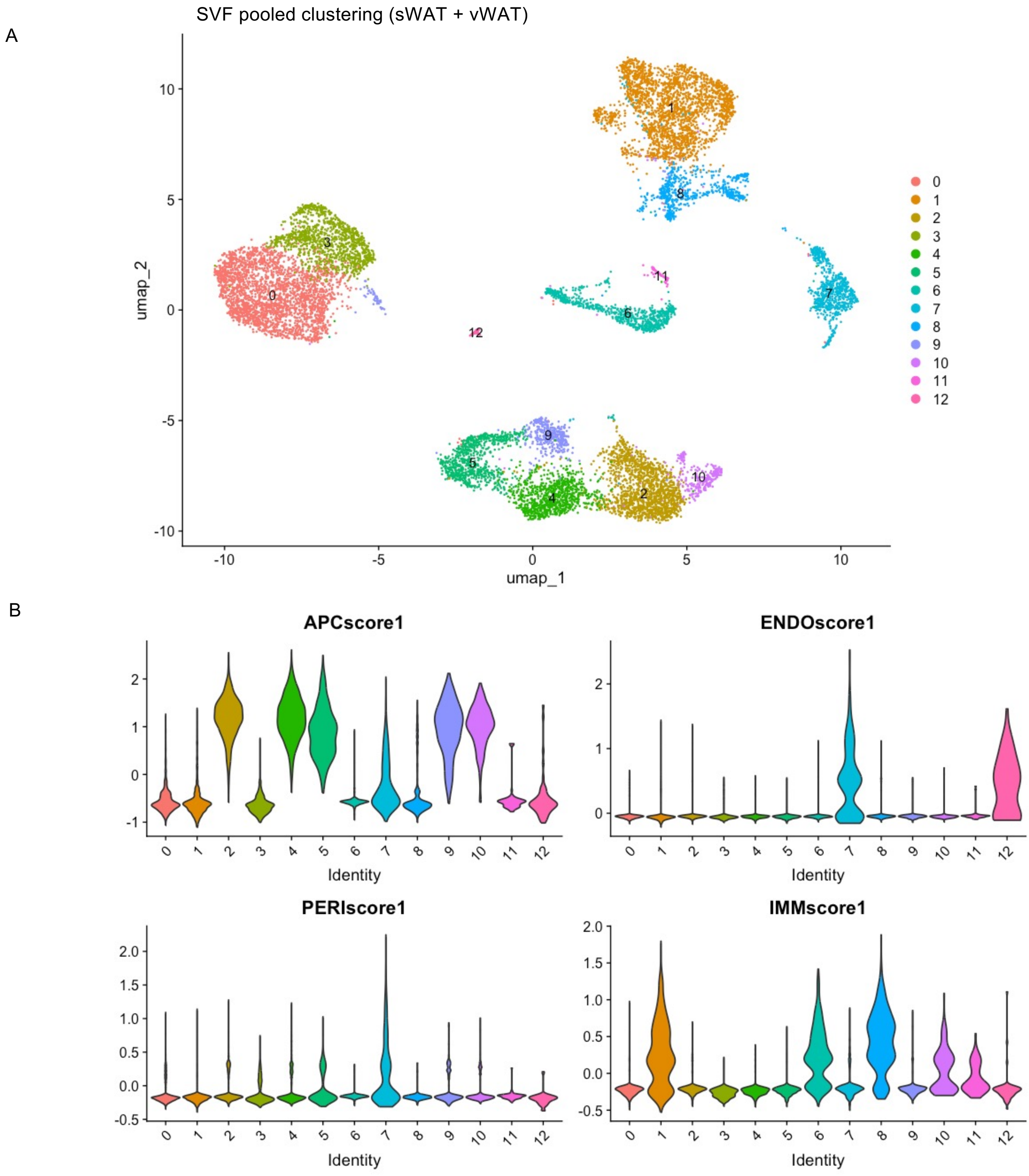

A
SVF pooled clustering (sWAT + vWAT)
umap_1
umap_2
0
1
2
3
4
5
6
7
8
9
10
11
12
B
APCscore1
ENDOscore1
PERIscore1
IMMscore1
Identity

Figure S4

C

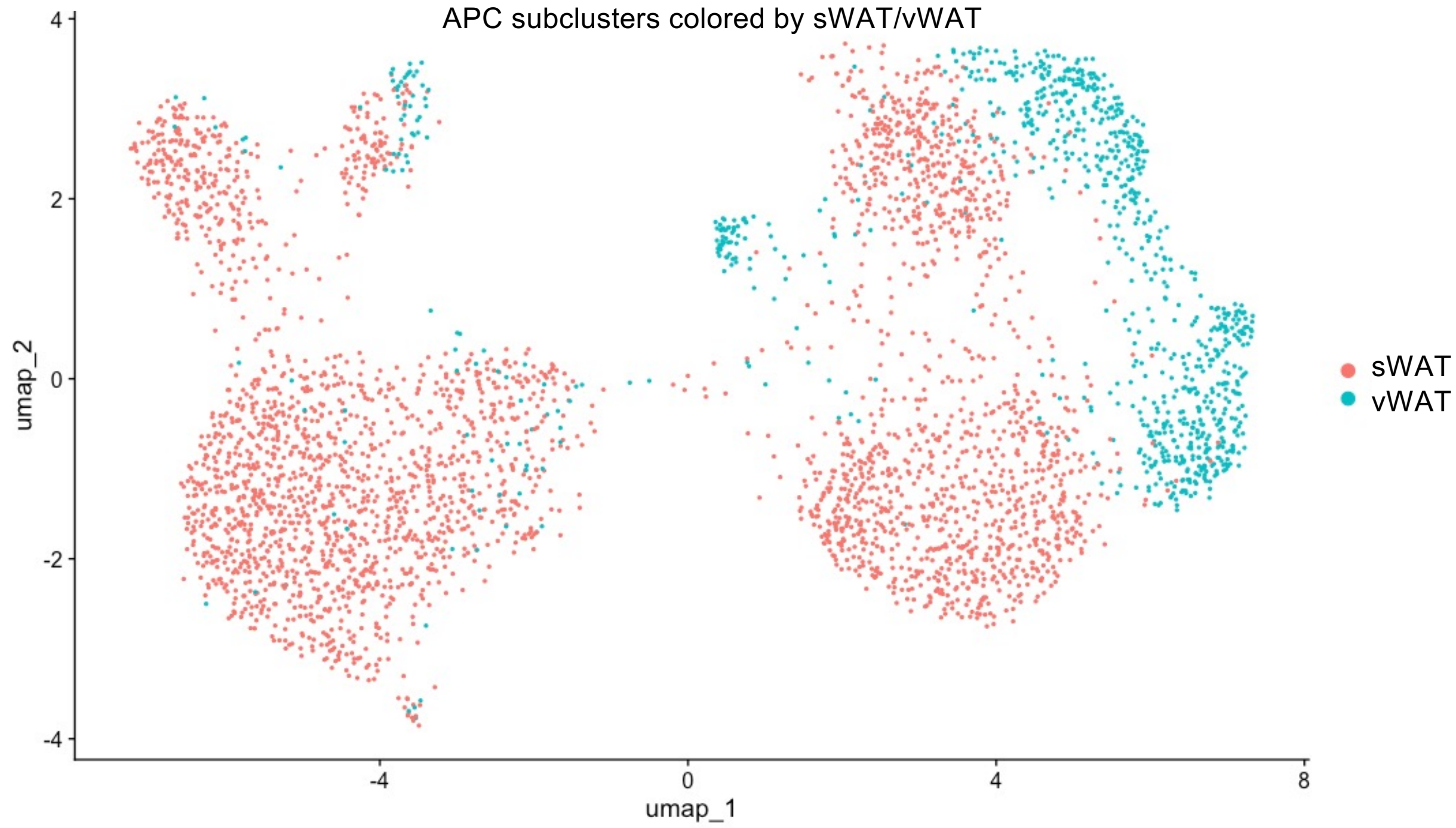

D

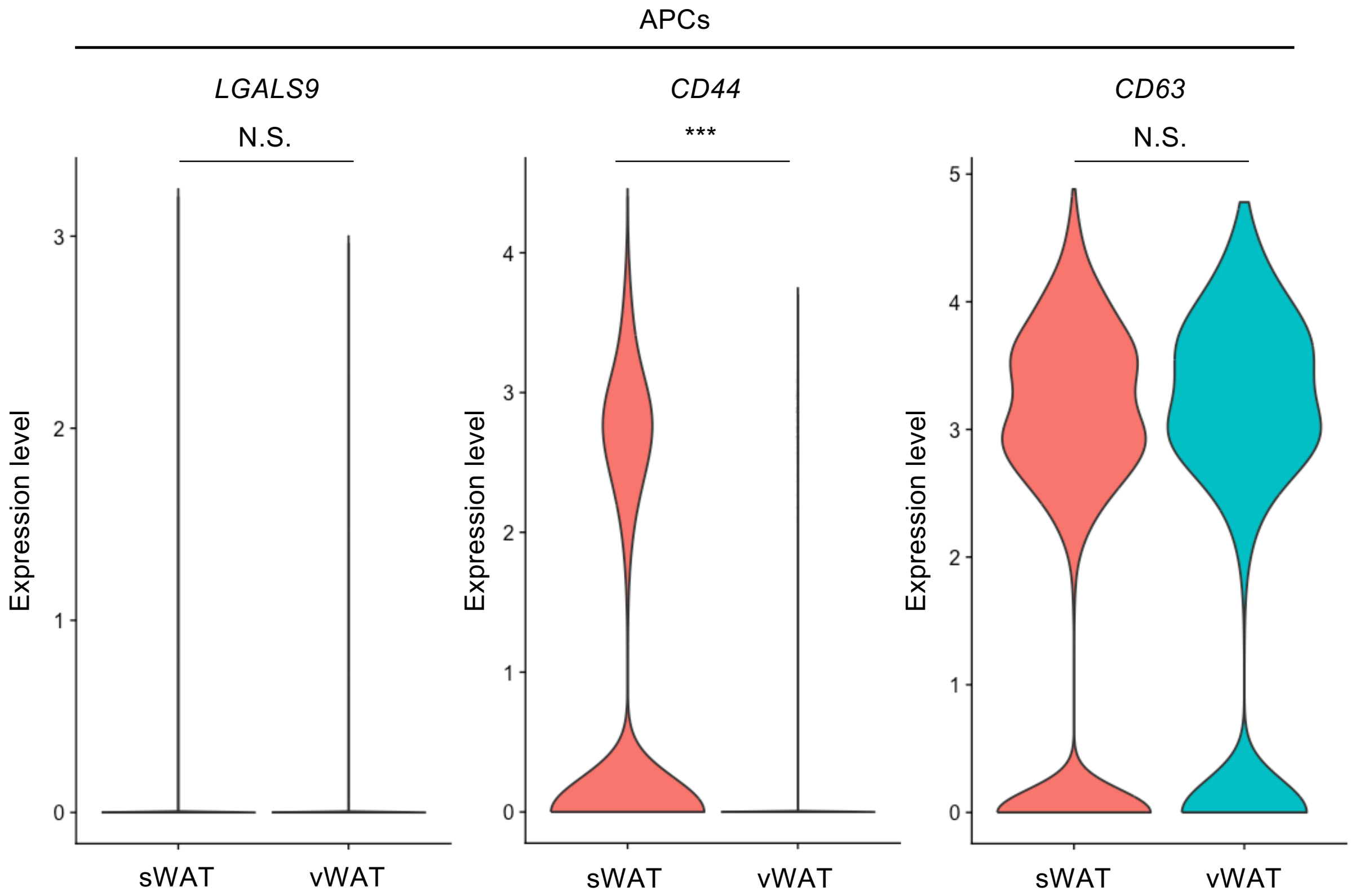

Figure S4. Reanalysis of publicly available scRNA-seq dataset (GSE129363).
(A) Uniform Manifold Approximation and Projection (UMAP) visualization of publicly available human stromal vascular fraction (SVF) single-cell RNA-seq data from subcutaneous (sWAT) and visceral (vWAT) adipose tissues. Cells are colored by unsupervised graph-based clustering. (B) Violin plots showing module scores for adipose progenitor cells (APC), endothelial cells (ENDO), pericytes (PERI), and immune cells (IMM) across SVF clusters. Module scores were calculated using canonical lineage marker gene sets as described in the Methods. (C) UMAP visualization of APC-enriched clusters re-clustered for higher-resolution analysis. Cells are colored by tissue of origin (red, sWAT; blue, vWAT). (D) Violin plots showing expression levels of LGALS9 ($P = 0.33$), CD44 ($P = 0.000002$), and CD63 ($P = 0.49$) in APC subclusters, stratified by tissue of origin (red, sWAT; blue, vWAT). Data were analyzed by Welch's t-test; ***$P < 0.001$.

Figure S5

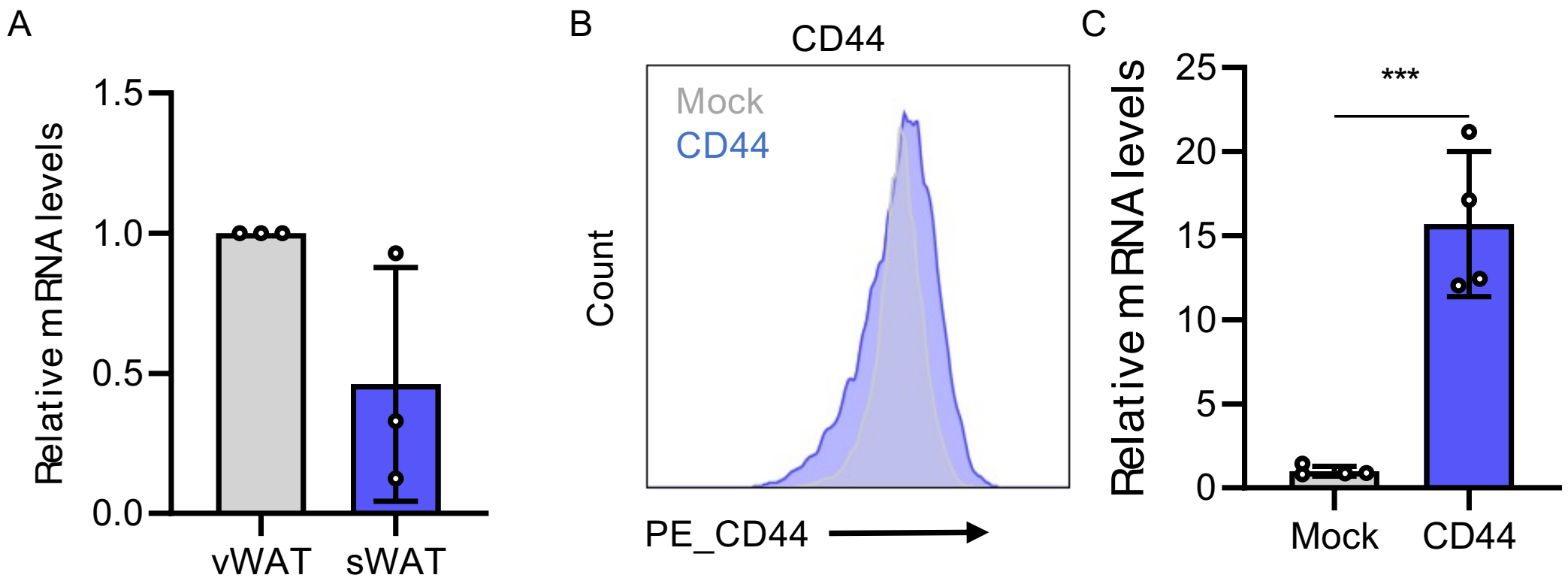

Figure S5. Additional data supporting Figure 3.
(A) CD44 mRNA levels in sWAT and vWAT. (B) Flow cytometry histogram confirming CD44 overexpression in 3T3-L1 cells using a PE-conjugated anti–CD44 antibody. (C) Cd44 mRNA levels in mock and CD44 OE 3T3-L1 cells (P = 0.0005). Data are expressed as the mean ± SD and were analyzed by Student's t-test; ***P < 0.001.

Figure S6

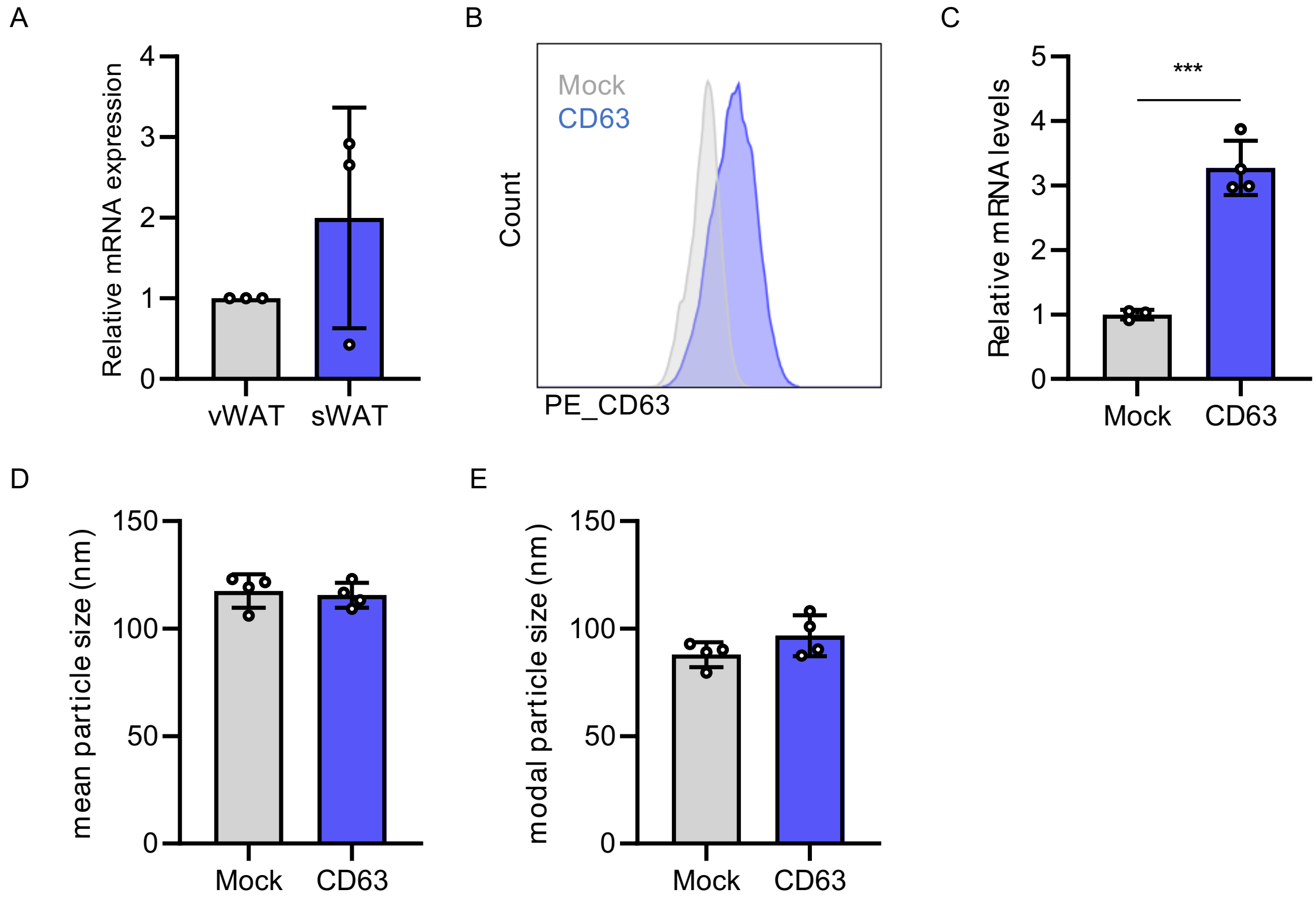

Figure S6. Additional data supporting Figure 4.
(A) CD63 mRNA levels in sWAT and vWAT. (B) Flow cytometry histograms showing CD63 expression in mock and CD63 OE 3T3-L1 cells stained with a PE-conjugated anti–CD63 antibody. (C) CD63 mRNA levels in mock and CD63 OE 3T3-L1 cells ($P = 0.0002$). (D) Mean and (E) modal extracellular vesicle particle size. Data are expressed as the mean ± SD and were analyzed by Student's t-test; *$P < 0.05$, **$P < 0.01$, ***$P < 0.001$.

Original blots are listed by figure numbers.
Highlighted regions correspond to the figures in manuscript.

Fig.2C

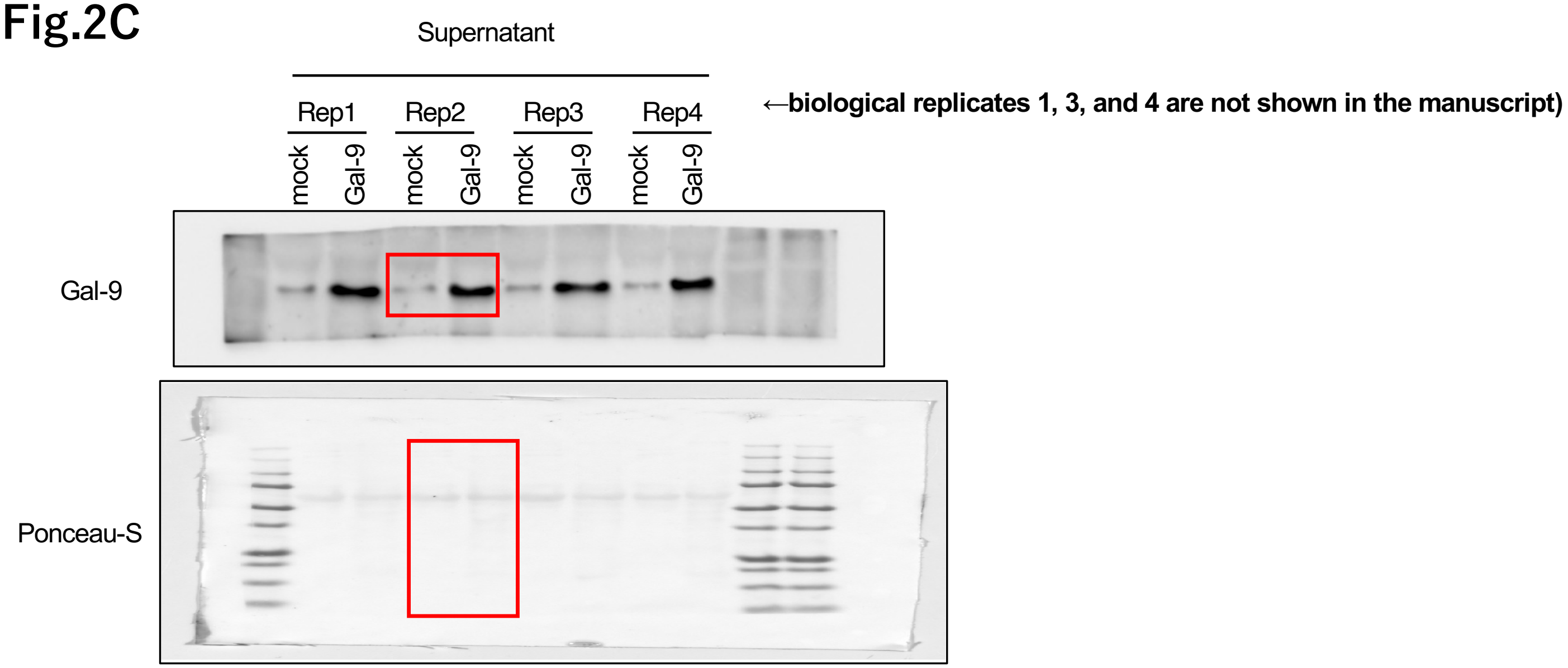
Supernatant
Rep1
Rep2
Rep3
Rep4
mock
Gal-9
mock
Gal-9
mock
Gal-9
mock
Gal-9
←biological replicates 1, 3, and 4 are not shown in the manuscript)
Gal-9
Ponceau-S

Fig.4D

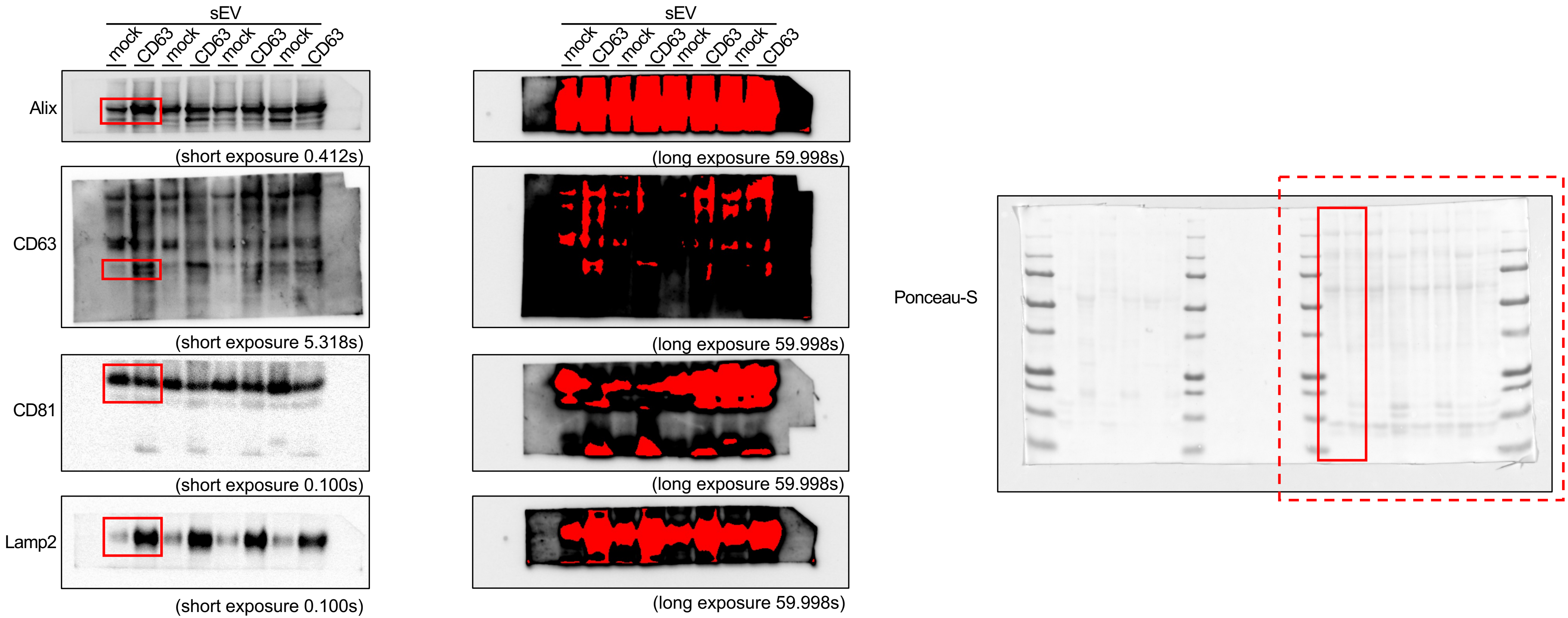
sEV
mock
CD63
mock
CD63
mock
CD63
mock
CD63
Alix
(short exposure 0.412s)
CD63
(short exposure 5.318s)
CD81
(short exposure 0.100s)
Lamp2
(short exposure 0.100s)
sEV
mock
CD63
mock
CD63
mock
CD63
mock
CD63
(long exposure 59.998s)
(long exposure 59.998s)
(long exposure 59.998s)
(long exposure 59.998s)
Ponceau-S

Figure 4D – Additional biological replicates (not shown in the manuscript)

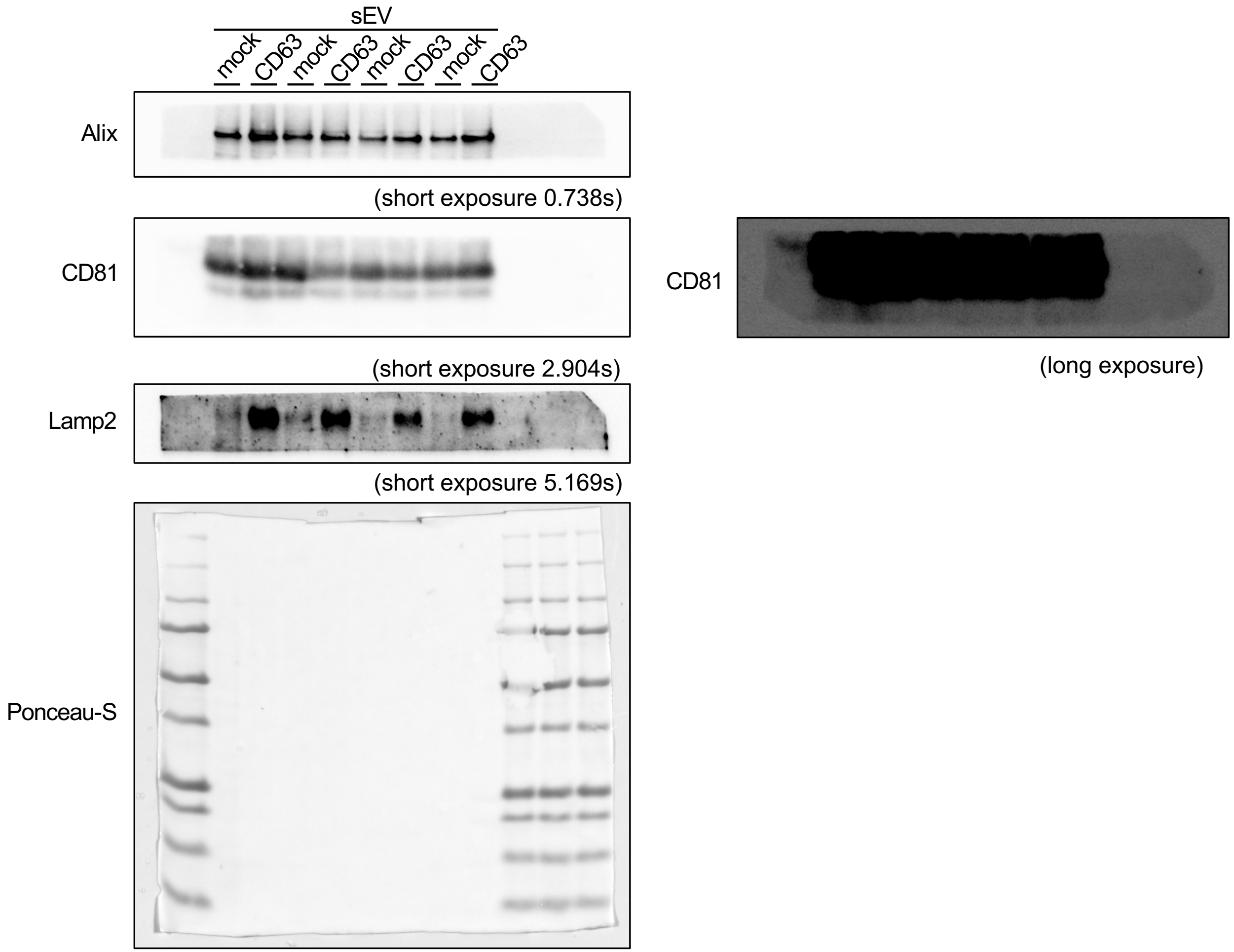

Figure 4D,F – Additional biological replicates (not shown in the manuscript)

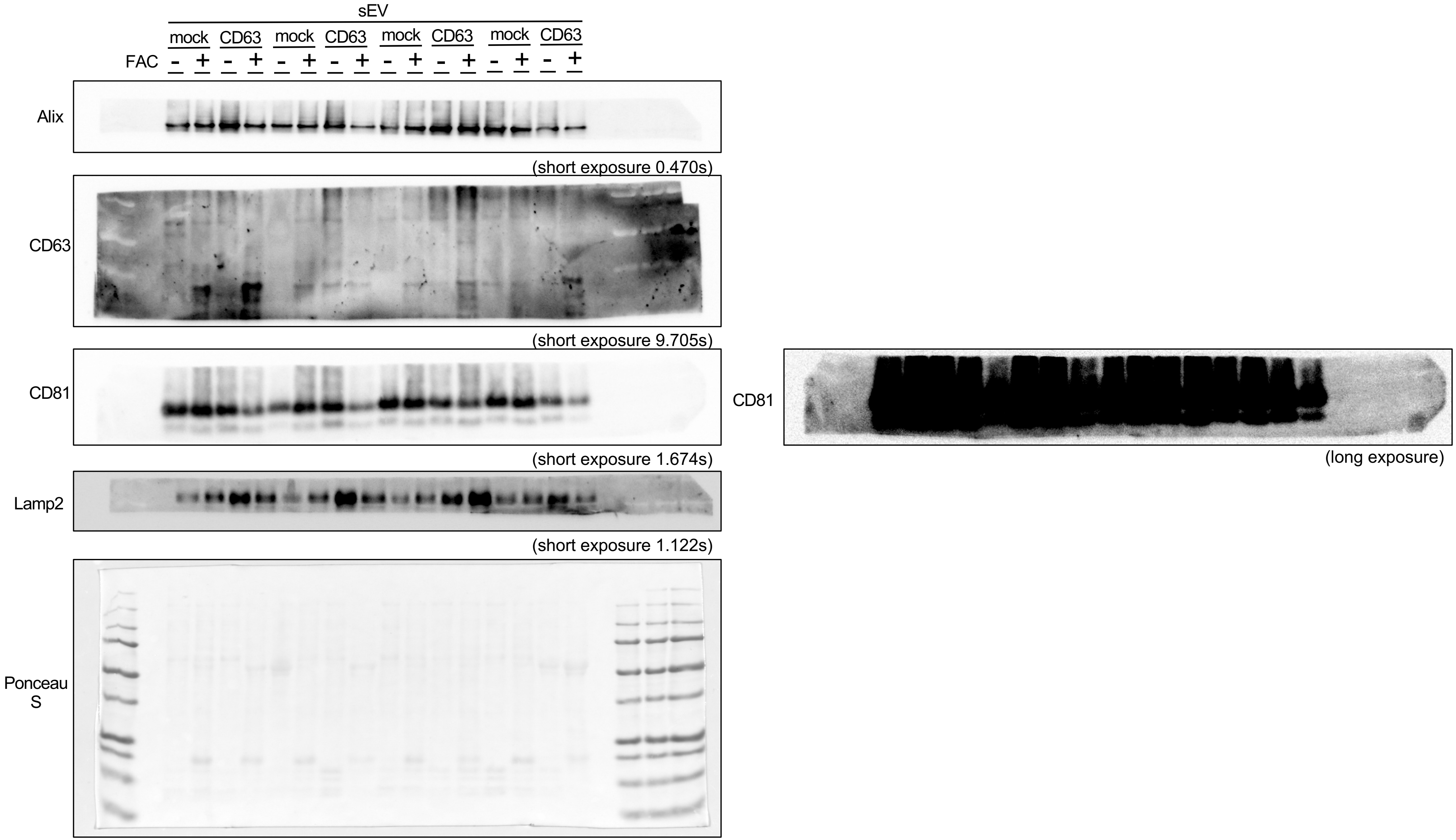

Figure 4D,F – Additional biological replicates (not shown in the manuscript)

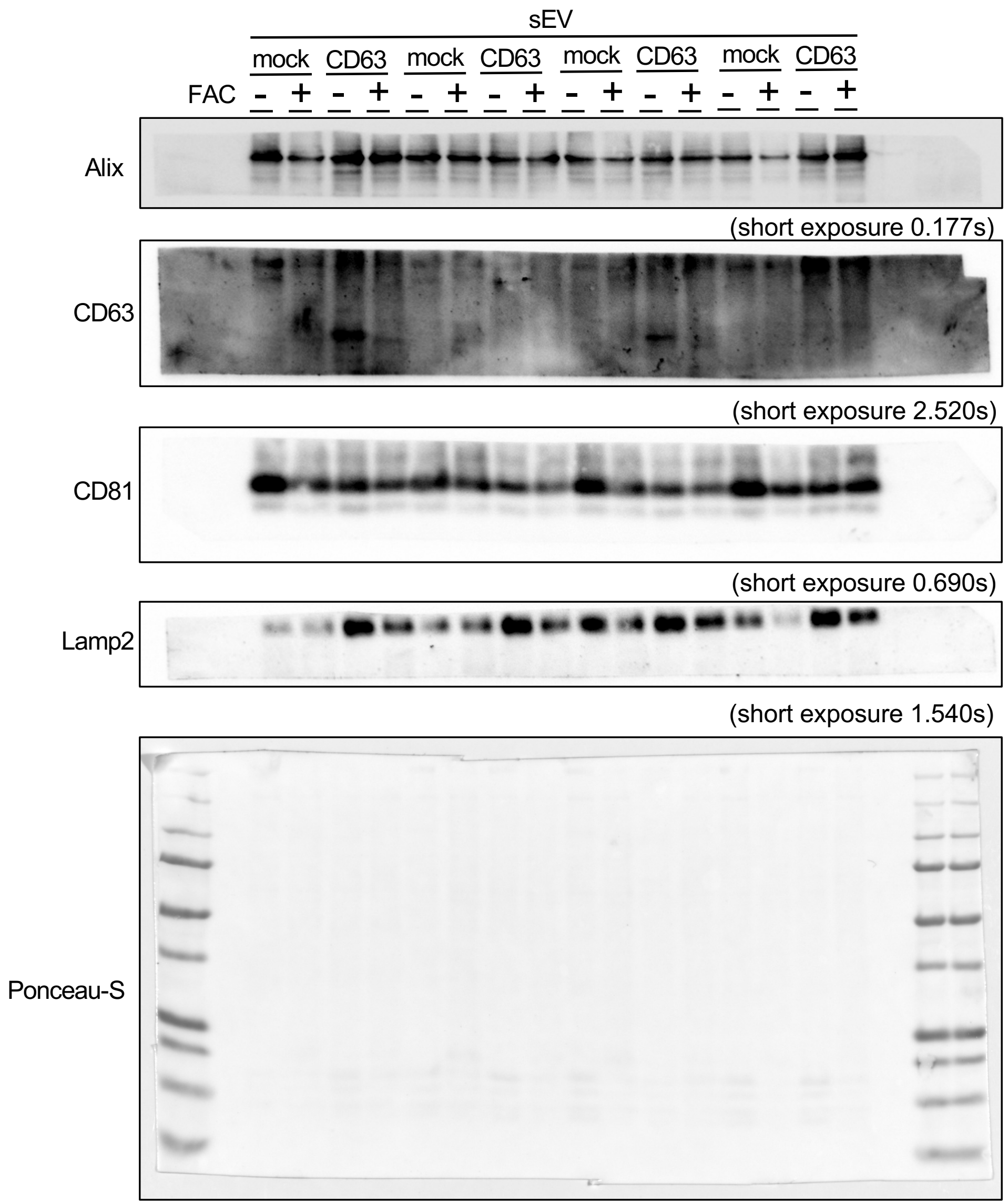

Fig.4F

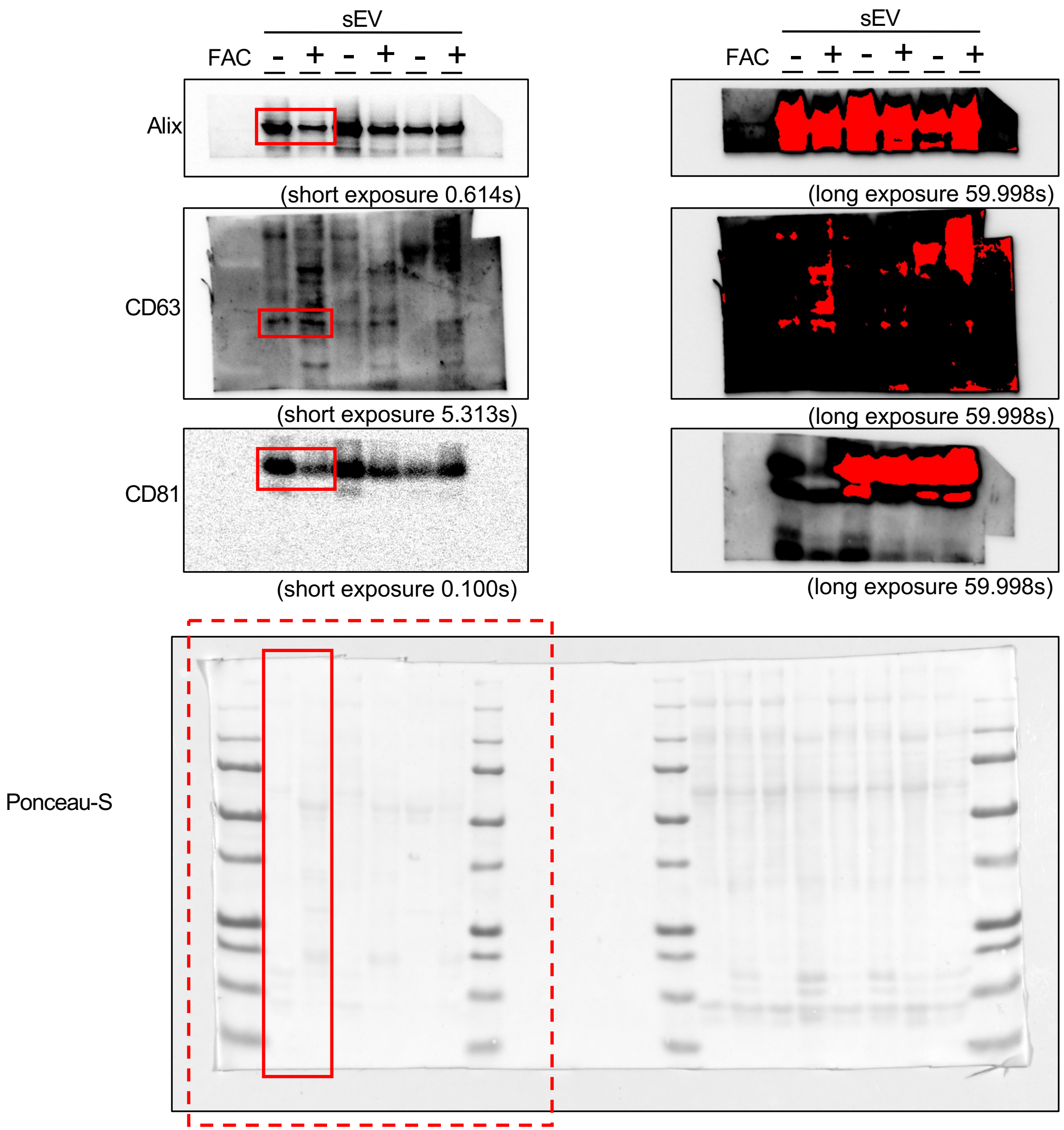

sEV
FAC - + - + - +
Alix
(short exposure 0.614s)
sEV
FAC - + - + - +
(long exposure 59.998s)
CD63
(short exposure 5.313s)
(long exposure 59.998s)
CD81
(short exposure 0.100s)
(long exposure 59.998s)
Ponceau-S
