# Supplementary material for: Surface protein profiling reveals depot-specific features of subcutaneous and visceral adipose progenitor cells
Source: Biochem Biophys Rep. 2026 Aug 17;47:102750. doi: 10.1016/j.bbrep.2026.102750 (PMC13507801; doi:10.1016/j.bbrep.2026.102750)
Supplement: Supplementary file 3 [file mmc3.pdf]

Original blots are listed by figure numbers.
Highlighted regions correspond to the figures in manuscript.

Fig.2C

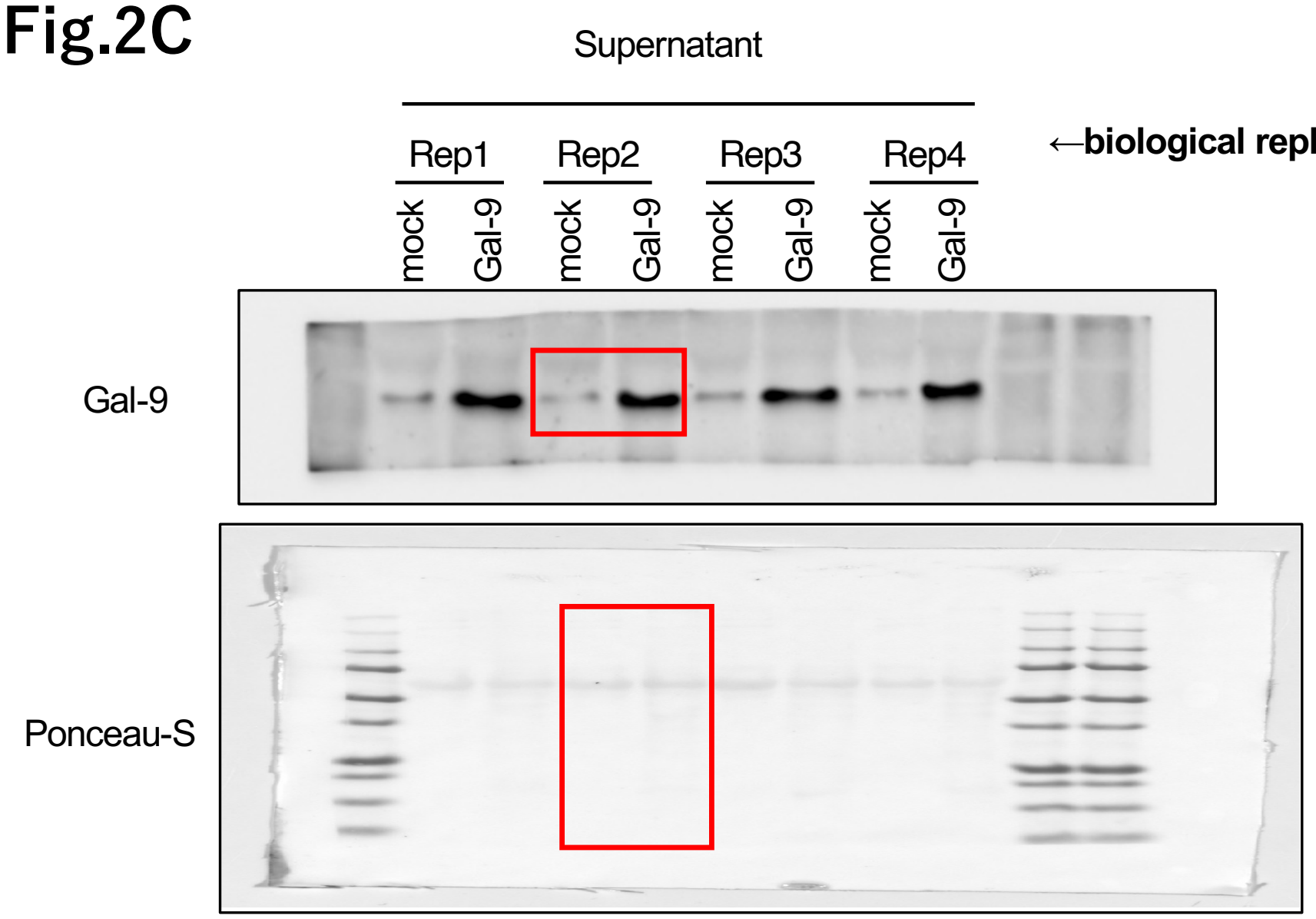

←biological replicates 1, 3, and 4 are not shown in the manuscript)

Fig.4D

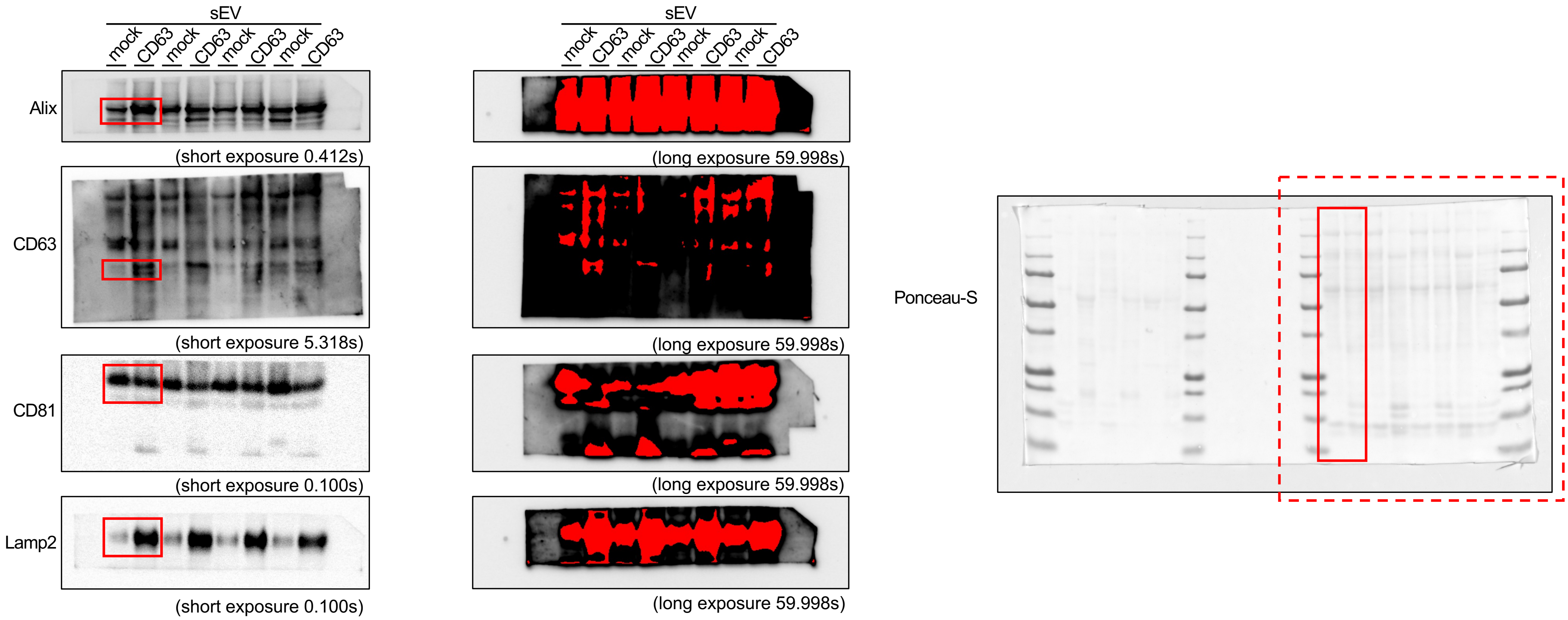
sEV
mock
CD63
mock
CD63
mock
CD63
mock
CD63
Alix
(short exposure 0.412s)
(long exposure 59.998s)
CD63
(short exposure 5.318s)
(long exposure 59.998s)
CD81
(short exposure 0.100s)
(long exposure 59.998s)
Lamp2
(short exposure 0.100s)
(long exposure 59.998s)
Ponceau-S

Figure 4D – Additional biological replicates (not shown in the manuscript)

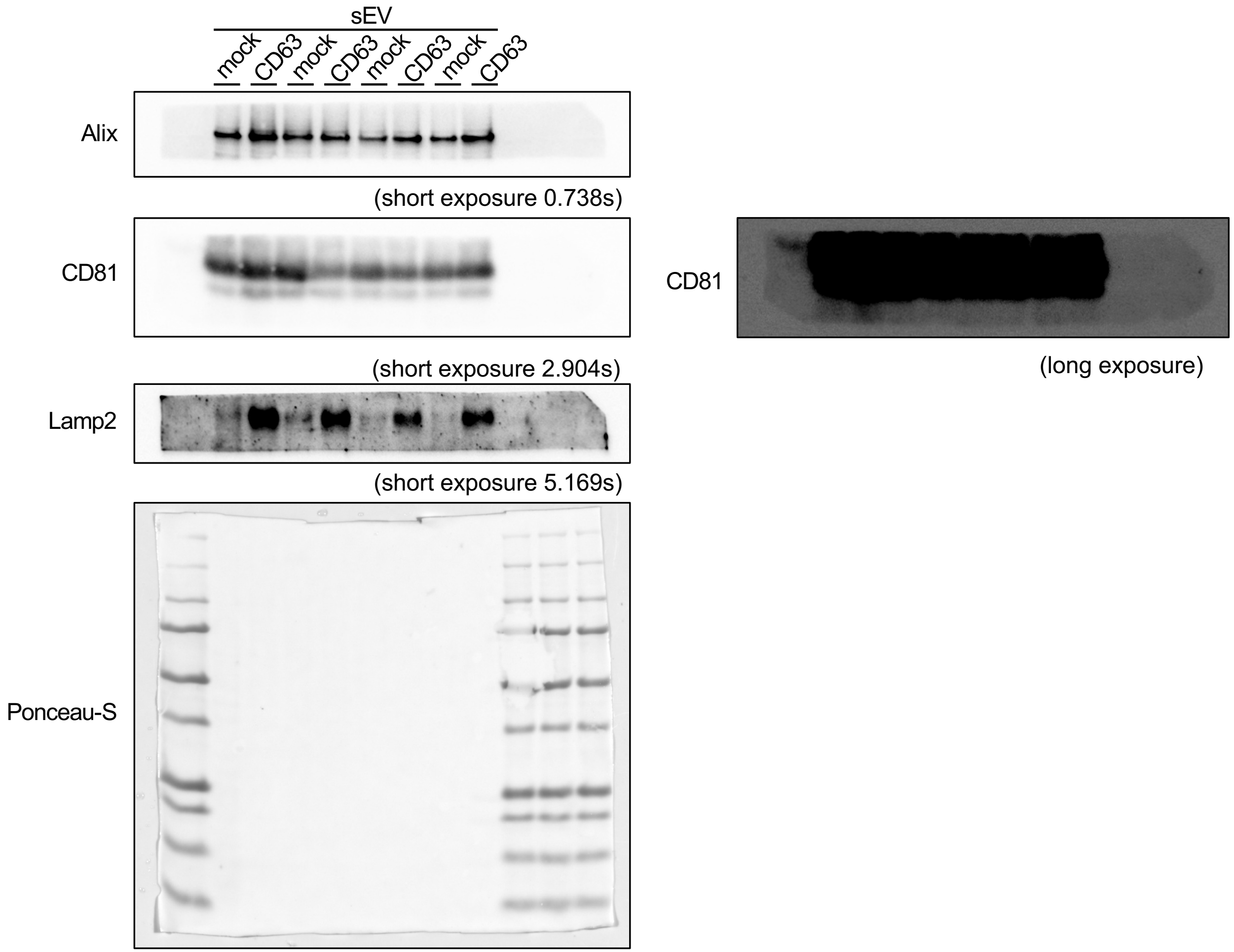

Figure 4D,F – Additional biological replicates (not shown in the manuscript)

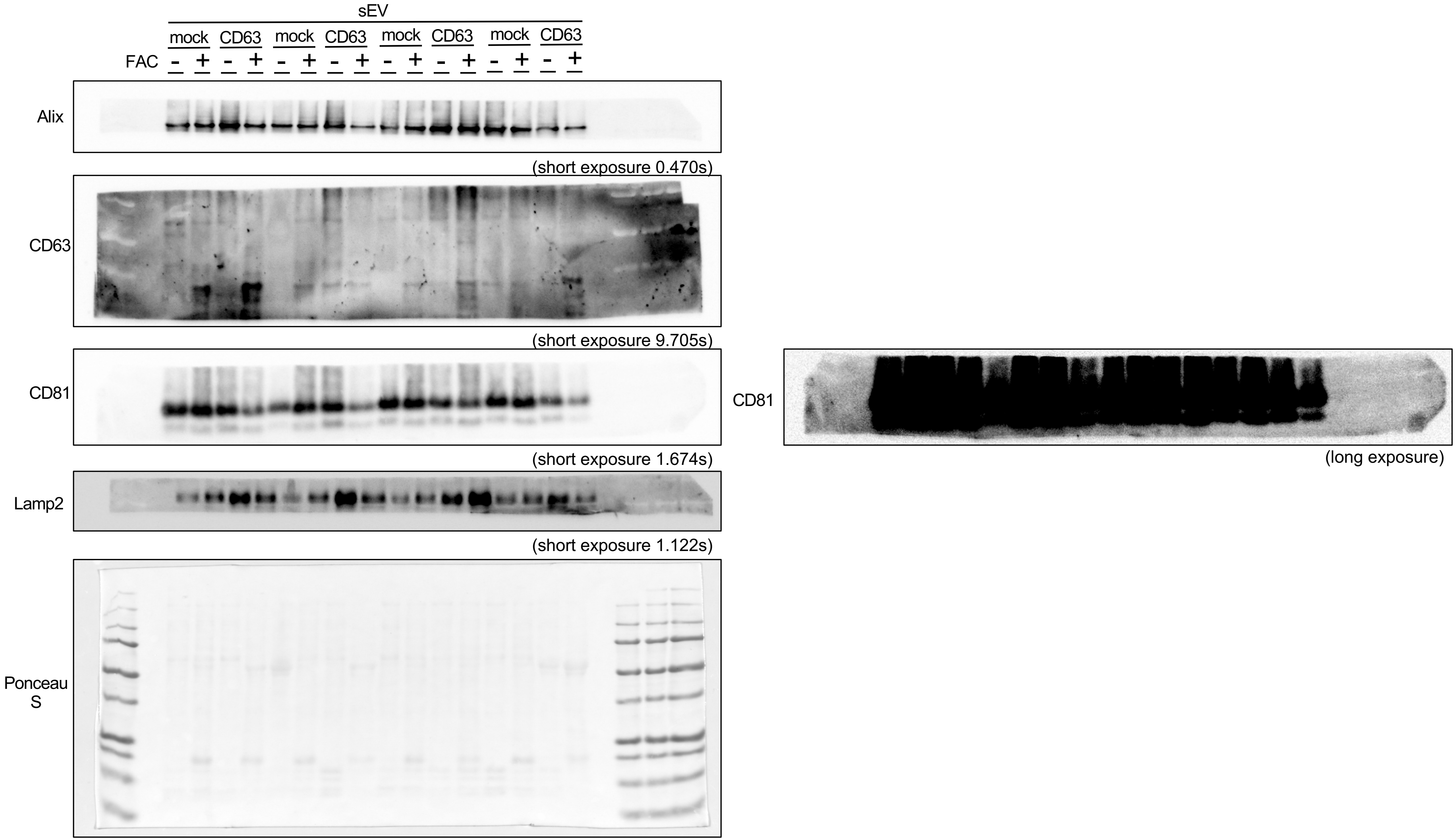

Figure 4D,F – Additional biological replicates (not shown in the manuscript)

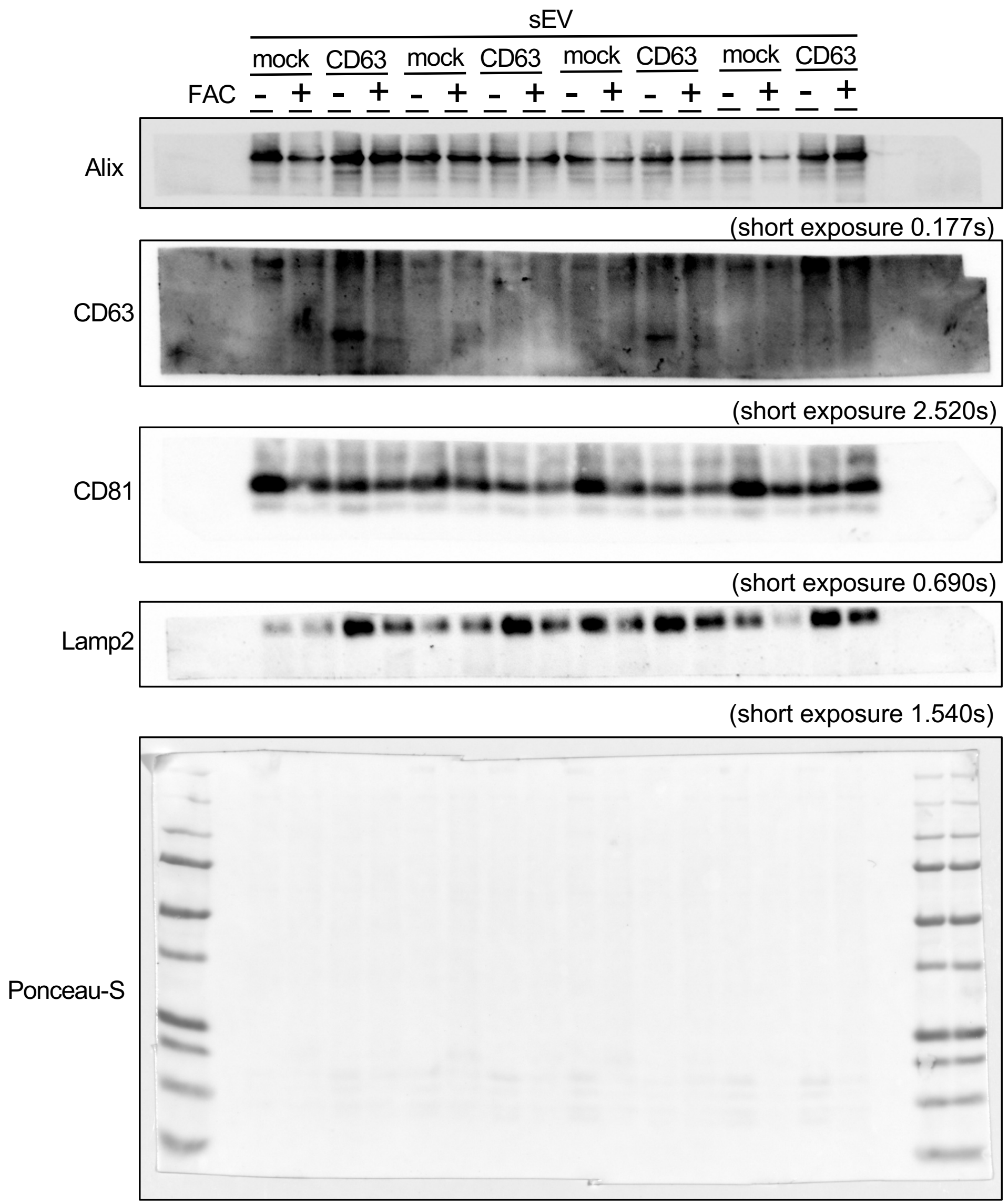

Fig.4F

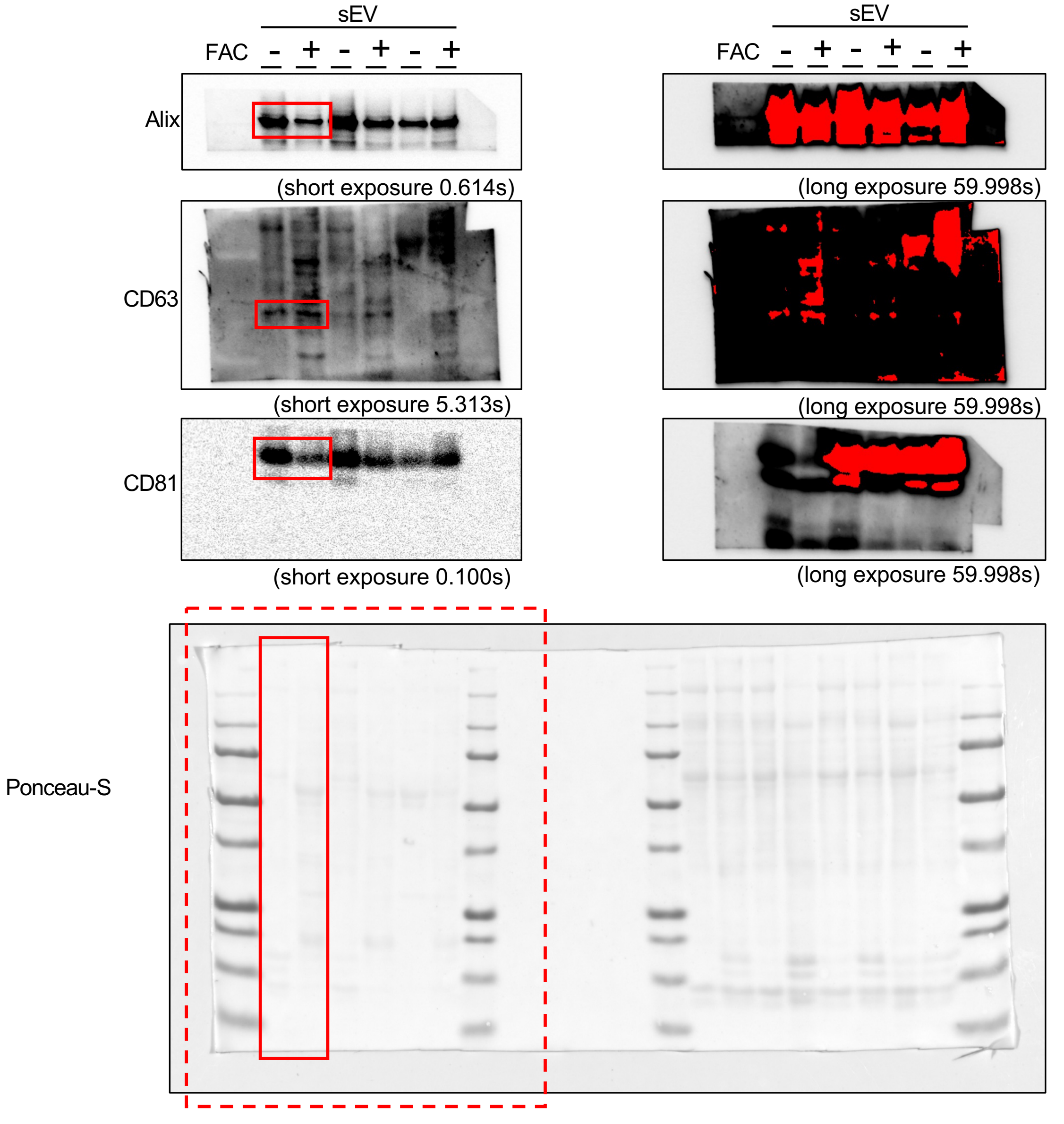
sEV
FAC - + - + - +
Alix
(short exposure 0.614s)
CD63
(short exposure 5.313s)
CD81
(short exposure 0.100s)
sEV
FAC - + - + - +
(long exposure 59.998s)
(long exposure 59.998s)
(long exposure 59.998s)
Ponceau-S
